# Supplementary material for: USP9X Limits Mitotic Checkpoint Complex Turnover to Strengthen the Spindle Assembly Checkpoint and Guard against Chromosomal Instability
Source: Cell Rep. 2018 Apr 17;23(3):852–65. doi: 10.1016/j.celrep.2018.03.100 (PMC5917450; doi:10.1016/j.celrep.2018.03.100)
Supplement: Document S1. Figures S1–S5 [file mmc1.pdf]

Cell Reports, Volume *23*

Supplemental Information

USP9X Limits Mitotic Checkpoint Complex Turnover to Strengthen the Spindle Assembly Checkpoint and Guard against Chromosomal Instability

Agnieszka Skowyra, Lindsey A. Allan, Adrian T. Saurin, and Paul R. Clarke

Figure S1. SiRNA-mediated depletion of USP9X weakens the SAC, Related to Figure 1.

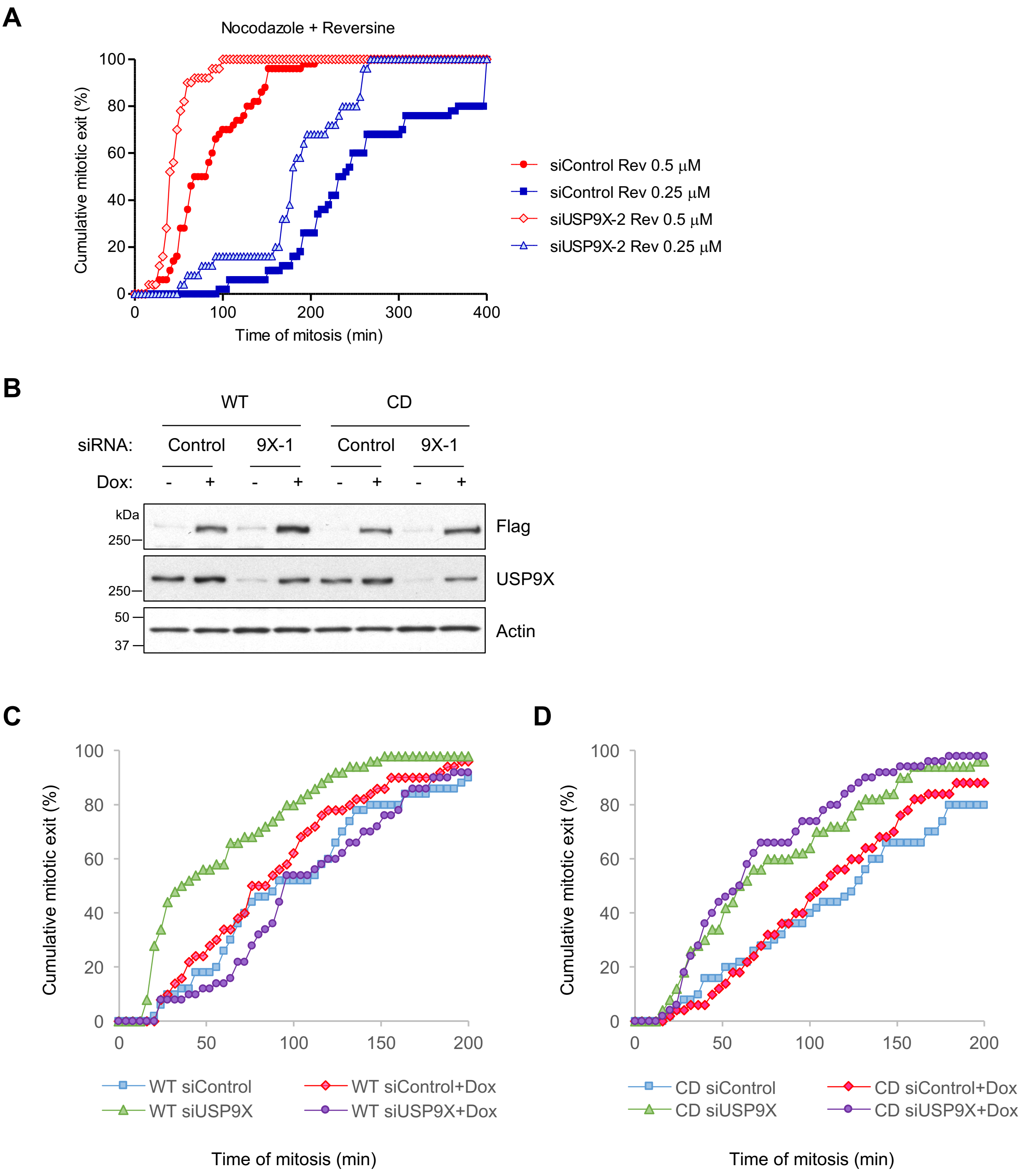

Figure S1. SiRNA-mediated depletion of USP9X weakens the SAC, Related to Figure 1.

A. Time-lapse analysis of duration of mitotic arrest in U2OS cells treated with control or USP9X-2 siRNAs and arrested in nocodazole with different concentrations of reversine, as indicated. 50 cells were analysed per condition, and graph is representative of 3 independent experiments.

B. Western blot showing induction of siRNA-resistant FLAG-USP9X wild type (WT) and catalytically dead (CD) protein after knockdown of endogenous USP9X in U2OS FRT cells. Cells were transfected with control or USP9X-1 siRNA for 24 h, prior to addition of Dox for 48 h to induce expression of Flag-USP9X. Samples were blotted with antibodies as indicated.

C,D. Time–lapse analysis of duration of mitosis in U2OS FRT cells expressing **(C)** Flag-USP9X wild type (WT) or (**D**) catalytically dead (CD) protein after knockdown of endogenous USP9X and induction of Flag-USP9X as in (**B**). Cells entered mitosis in the presence of nocodazole (250 ng/ml) and reversine (0.35 µM) and were imaged every 4 min. 50 cells were analysed per condition.

Figure S2. USP9X depletion does not affect the MPS1 or Aurora B activity at kinetochores, Related to Figure 2.

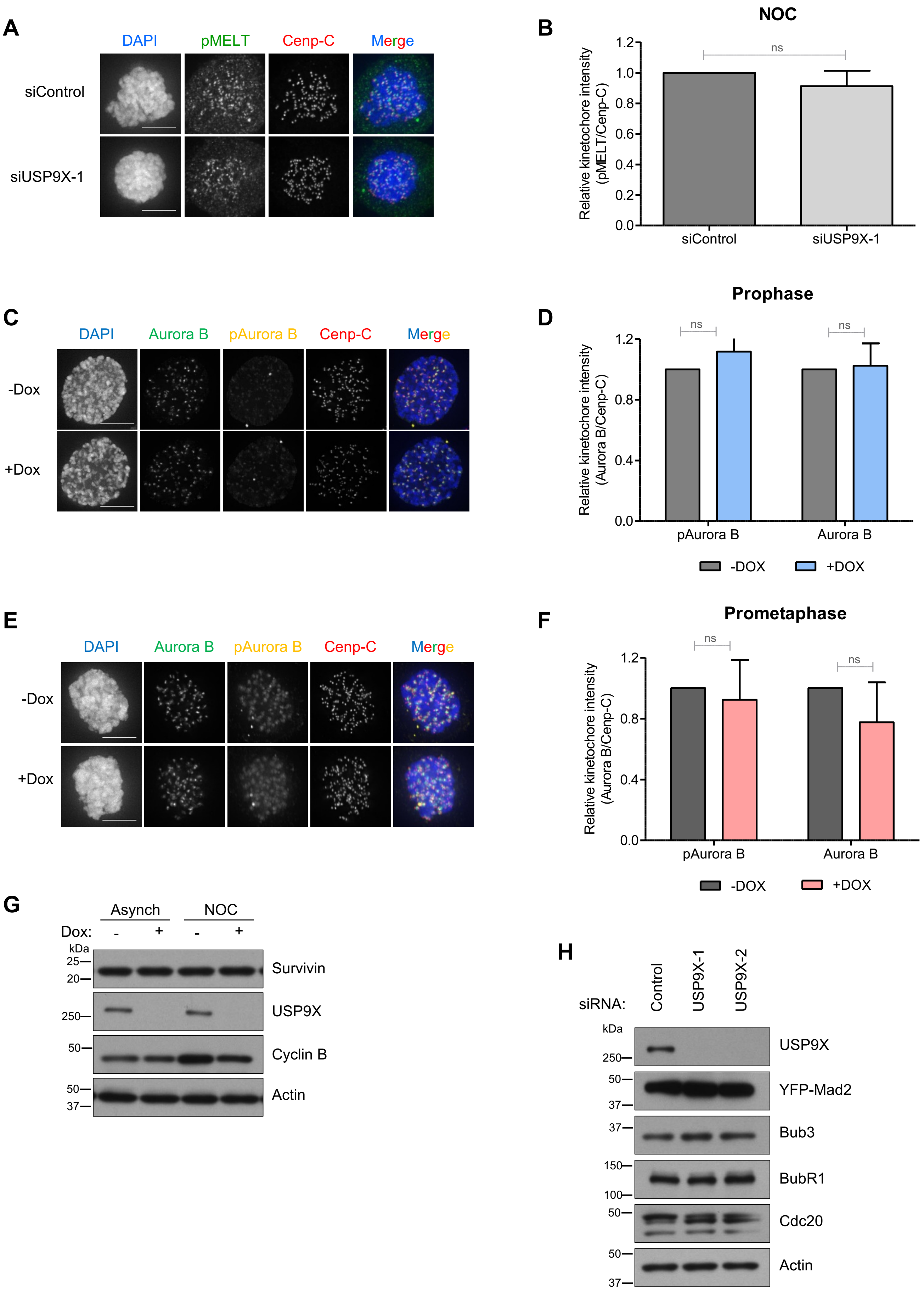

Figure S2. USP9X depletion does not affect the MPS1 or Aurora B activity at kinetochores, Related to Figure 2.

A,B. Representative images (A) and quantification (B) of KNL1-pMELT localization at kinetochores in nocodazole-arrested U2OS cells treated with control or USP9X siRNA. CENP-C was used as a kinetochore marker. Scale bar = 10 μM.

C-F. Representative images (C, E) and quantification (D, F) of phosphorylated Aurora B (Thr232) and total Aurora B localization at kinetochores during prophase (C, D) or prometaphase (E, F) in nocodazole-arrested HeLa cells that were uninduced (-Dox) or induced (+Dox) to express USP9X shRNAs.

All graphs show the mean kinetochore intensities (± S.D.), relative to mock-transfected cells, from 3 independent experiments with 10 cells quantified for each condition per experiment. ns, non-significant, Students unpaired t-test. Scale bar = 10 μM.

G. Western blot of whole-cell lysates, demonstrating equal levels of Survivin in cells treated as in (A).

H. Western blot of whole-cell lysates, demonstrating equal levels of MCC components in YFP-Mad2 expressing cells, transfected with control or USP9X siRNAs. Expression of YFP-Mad2 was induced 16 h before harvesting cells.

Figure S3. USP9X depletion does not affect the SAC-independent degradation of APC/C substrates, Related to Figure 3.

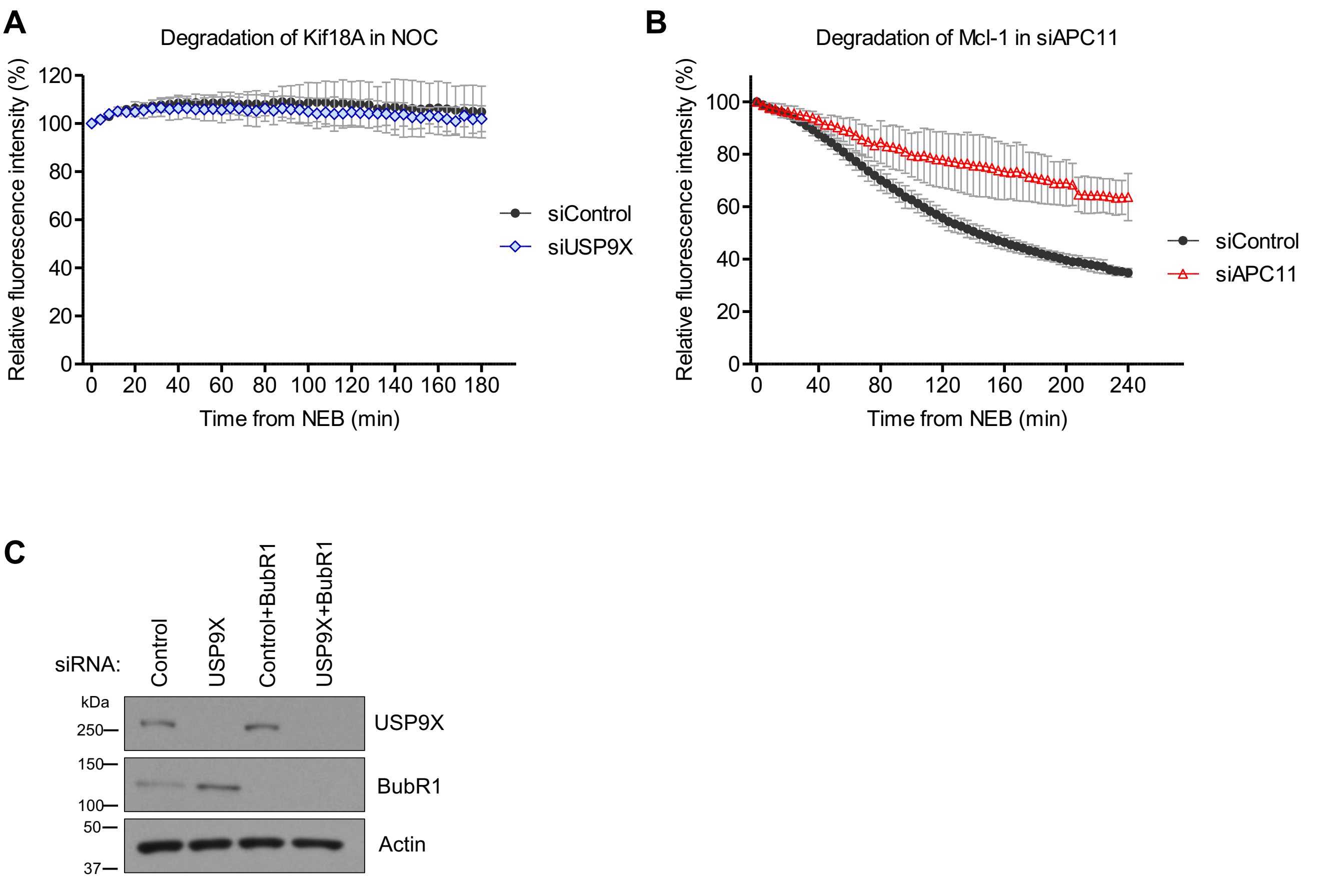

Figure S3. USP9X depletion does not affect the SAC-independent degradation of APC/C substrates, Related to Figure 3.

A,B. Time-lapse analysis of Venus-Kif18A degradation (A) or YFP-Mcl-1 degradation (B) in HeLa cells treated with control siRNA compared to either USP9X siRNA or APC11 siRNA, respectively, and arrested in mitosis with nocodazole. Graphs show the mean values (± S.D.) from 3 independent experiments with 10 cells quantified for each condition per experiment.

C. Western blot of whole-cell lysates of U2OS cells expressing endogenous Cyclin B-eYFP, demonstrating co-depletion of USP9X and BubR1.

Figure S4. Effect of USP9X depletion on APC/C-mediated CDC20 degradation and MCC turnover, Related to Figure 4.

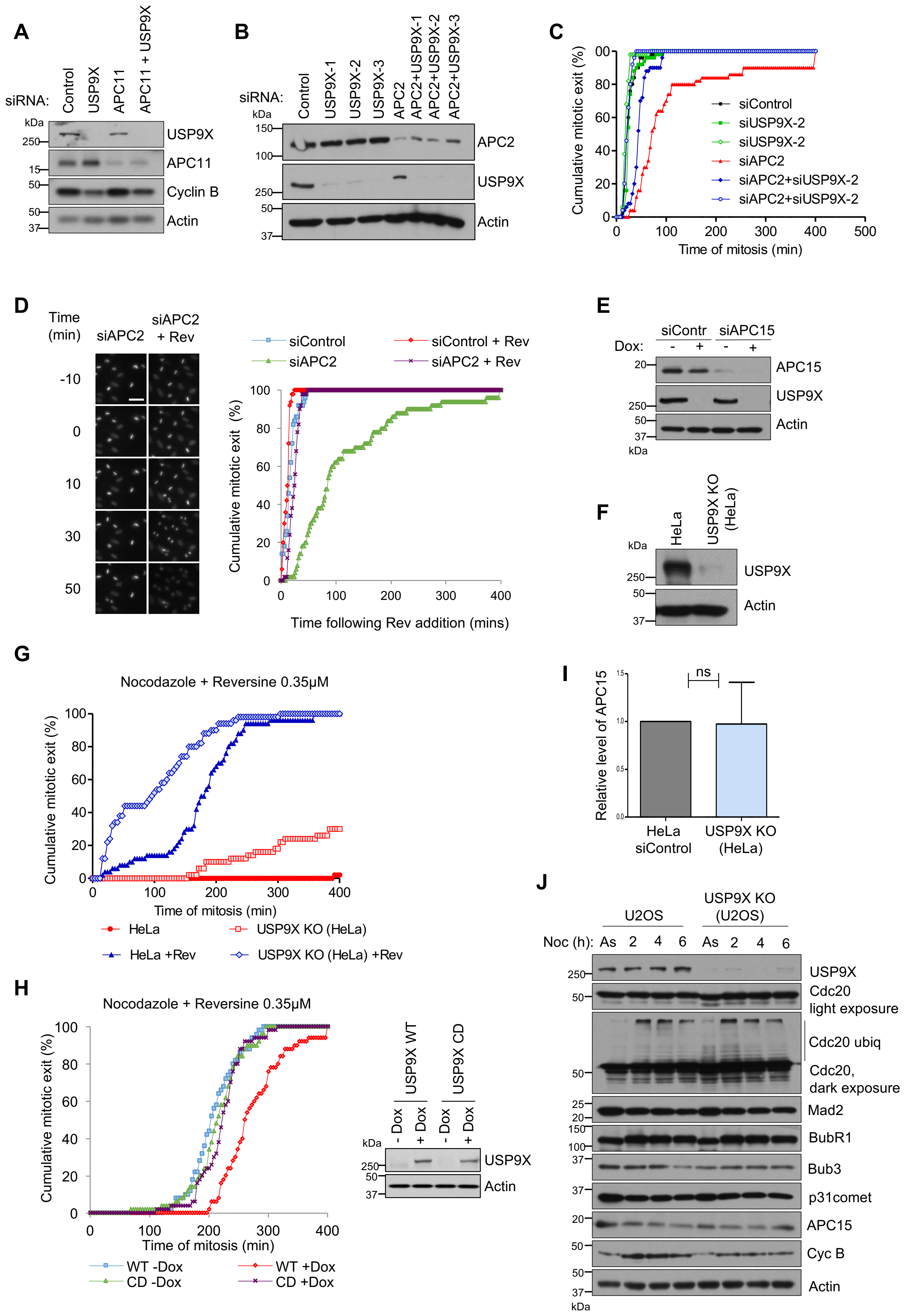

Figure S4. Effect of USP9X depletion on APC/C-mediated CDC20 degradation and MCC turnover, Related to Figure 4.

A. Western analysis demonstrating knockdown of USP9X and partial knockdown of APC11 in U2OS cells.

B. Western blot of whole-cell lysates from U2OS cells treated with indicated siRNAs, demonstrating knockdown of USP9X and partial knockdown of APC2.

C. Time-lapse analysis of duration of mitotic arrest in U2OS cells treated with indicated siRNAs. The graph shows cumulative data from at least 50 cells from one experiment, which is representative of 3 independent experiments.

D. Representative images and time-lapse analysis of duration of mitosis in HeLa-FRT cells transfected with control or APC2 siRNAs and allowed to arrest at metaphase. Cell were then either left untreated or treated with reversine (1µM) at t=0 min. The graph shows duration of mitosis following reversine addition. Scale bar = 50 µM.

E. Western analysis from HeLa cells, non-induced (-) or induced (+) to express USP9X shRNAs and treated with control or APC15 siRNAs, demonstrating APC15 knockdown is unaffected by USP9X co-depletion.

F. Western blot analysis demonstrating loss of USP9X protein in USP9X knockout cells compared with parental HeLa cells.

G. Time-lapse analysis of duration of mitotic arrest in control or USP9X knockout HeLa cells arrested in nocodazole with and without reversine (0.35 µM). The graph shows cumulative data from at least 50 cells from one experiment, which is representative of 2 independent experiments.

H. Time-lapse analysis of duration of mitotic arrest in HeLa USP9X knockout cells expressing doxycycline-inducible -wild-type (WT) or -catalytically dead (CD) USP9X. Cells were untreated or treated with Dox (1µg/ml) for 48h. Nocodazole (250ng/ml) and reversine (0.35µM) were added prior to imaging every 4 min. Dox-induction of WT- or CD- USP9X was analysed by western blotting. The graph shows cumulative data from at least 50 cells from one experiment, which is representative of 3 independent experiments.

I. Quantification of APC15 protein levels in parental and USP9X knockout (USP9X KO) HeLa cells using actin levels to control for loading. The graph shows the mean values (± S.D.) from 3 independent experiments. ns, non-significant, Students t-test.

J. Western blot analysis of MCC components and proteins involved in MCC turnover from control or USP9X knockout U2OS cells, either asynchronously growing (As) or arrested in nocodazole for indicated durations. Nocodazole-arrested cells were detached by shake off to exclude non-mitotic cells. Note increased ubiquitination of Cdc20 in nocodazole-arrested USP9X knockout (USP9X KO) cells compared to U2OS FRT cells with USP9X (U2OS).

Figure S5. Characterisation of HeLa-CRISPR-Cas9 USP9X knockout cells, Related to Figure 5.

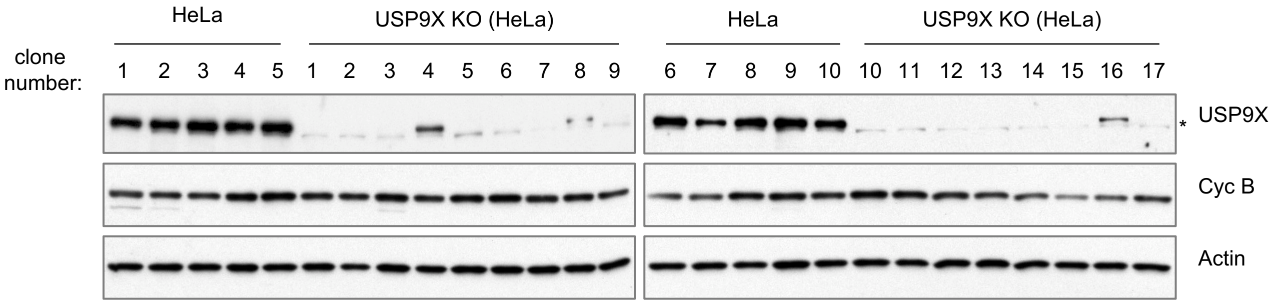

Figure S5. Characterisation of HeLa-CRISPR-Cas9 USP9X knockout cells, Related to Figure 5. Western blotting showing absence of USP9X protein in USP9X knock-out (USP9X KO) single clones. Parental HeLa-Flp-in clones were analysed for comparison. * denotes a non-specific band detected by the USP9X antibody.
