# Supplementary material for: USP9X Limits Mitotic Checkpoint Complex Turnover to Strengthen the Spindle Assembly Checkpoint and Guard against Chromosomal Instability
Source: Cell Rep. 2018 Apr 17;23(3):852–65. doi: 10.1016/j.celrep.2018.03.100 (PMC5917450; doi:10.1016/j.celrep.2018.03.100)
Supplement: Document S2. Article plus Supplemental Information [file mmc2.pdf]

Cell Reports

Article

USP9X Limits Mitotic Checkpoint Complex Turnover to Strengthen the Spindle Assembly Checkpoint and Guard against Chromosomal Instability

Graphical Abstract

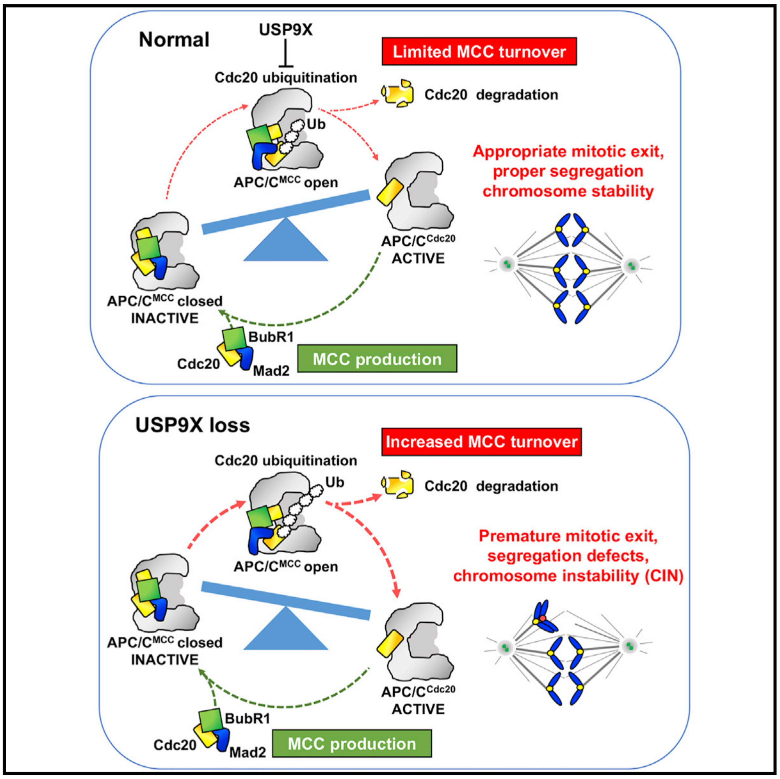

Highlights

- The deubiquitinase USP9X reinforces the spindle assembly checkpoint
- USP9X controls the mitotic checkpoint complex (MCC) downstream of kinetochores
- USP9X inhibits the ubiquitination and degradation of Cdc20, a component of MCC
- USP9X loss elevates chromosomal instability (CIN)

Authors

Agnieszka Skowyra, Lindsey A. Allan, Adrian T. Saurin, Paul R. Clarke

Correspondence

a.saurin@dundee.ac.uk (A.T.S.),
paul.clarke@uq.edu.au (P.R.C.)

In Brief

Skowyra et al. show the deubiquitinase USP9X limits activation of the ubiquitin ligase APC/C during mitosis. Loss of USP9X causes chromosomal instability (CIN), which can promote cancer. This work also provides a rationale for targeting USP9X when it is expressed in cancer cells with high levels of CIN.

Skowyra et al., 2018, Cell Reports *23*, 852–865
April 17, 2018 © 2018 The Author(s).
https://doi.org/10.1016/j.celrep.2018.03.100

CellPress

OPEN ACCESS
CellPress

Cell Reports
Article

USP9X Limits Mitotic Checkpoint Complex Turnover to Strengthen the Spindle Assembly Checkpoint and Guard against Chromosomal Instability

Agnieszka Skowyra,[1] Lindsey A. Allan,[1] Adrian T. Saurin,[1,3,*] and Paul R. Clarke[1,2,3,*]
[1]Division of Cancer Research, School of Medicine, University of Dundee, Jacqui Wood Cancer Centre, Ninewells Hospital and Medical School, Dundee DD1 9SY, UK
[2]The University of Queensland Diamantina Institute, Faculty of Medicine, Translational Research Institute, 37 Kent Street, Woolloongabba QLD 4102, Australia
[3]Lead Contact
*Correspondence: a.saurin@dundee.ac.uk (A.T.S.), paul.clarke@uq.edu.au (P.R.C.)
https://doi.org/10.1016/j.celrep.2018.03.100

SUMMARY

Faithful chromosome segregation during mitosis depends on the spindle assembly checkpoint (SAC), which delays progression through mitosis until every chromosome has stably attached to spindle microtubules via the kinetochore. We show here that the deubiquitinase USP9X strengthens the SAC by antagonizing the turnover of the mitotic checkpoint complex produced at unattached kinetochores. USP9X thereby opposes activation of anaphase-promoting complex/cyclosome (APC/C) and specifically inhibits the mitotic degradation of SAC-controlled APC/C substrates. We demonstrate that depletion or loss of USP9X reduces the effectiveness of the SAC, elevates chromosome segregation defects, and enhances chromosomal instability (CIN). These findings provide a rationale to explain why loss of USP9X could be either pro- or anti-tumorigenic depending on the existing level of CIN.

INTRODUCTION

During mitosis, the spindle assembly checkpoint (SAC) safeguards against aneuploidy by ensuring that each and every chromosome is stably attached to microtubules before anaphase is allowed to proceed (Lischetti and Nilsson, 2015; Musacchio, 2015). Microtubules attach to each chromosome via the kinetochore, a large multiprotein complex assembled on centromeres (Pesenti et al., 2016). When kinetochores are not attached to microtubules, they provide a platform for assembly of the mitotic checkpoint complex (MCC), which inhibits the anaphase-promoting complex/cyclosome (APC/C), an E3 ubiquitin ligase required to initiate mitotic exit (Alfieri et al., 2016; Yamaguchi et al., 2016). The MCC is composed of the APC/C co-activator Cdc20, bound to Mad2, BubR1, and Bub3 (Chao et al., 2012). All of these components accumulate at unattached kinetochores, and a key event in MCC assembly is the ability of kinetochore-bound Mad1 to catalyze the conversion of "open" Mad2 into "closed" Mad2 that can then stably bind to Cdc20 (Luo et al., 2002; Sironi et al., 2002). The mature MCC can diffuse throughout the cytoplasm and inhibit APC/C^{Cdc20} complexes to restrain progression through mitosis (Izawa and Pines, 2015).

The SAC has key properties of being both exquisitely sensitive and rapidly responsive. A single unattached kinetochore is able to generate enough signal to prevent anaphase, and yet this signal can still be silenced quickly after the last kinetochore has attached to microtubules (Rieder et al., 1995). This rapid silencing is a combination of fast signal termination at kinetochores, which shuts down new MCC production, and rapid turnover of APC/C-bound MCC in the cytoplasm (Etemad and Kops, 2016; Mansfeld et al., 2011). APC/C activity is critical for this MCC turnover, and the presence of the APC15 subunit, in particular, allows APC/C^{MCC} to adopt an open conformation that is able to auto-ubiquitinate and degrade MCC-bound Cdc20 (Alfieri et al., 2016; Foster and Morgan, 2012; Mansfeld et al., 2011; Uzunova et al., 2012). This MCC turnover is also aided by a second pathway involving p31^{comet} and TRIP13, which extracts Mad2 out of the MCC complex (Corbett, 2017).

The constant turnover of MCC is balanced during prometaphase by the continual synthesis of Cdc20 (Liang et al., 2012), and therefore, as long as unattached kinetochores are present, this can be incorporated into new MCC complexes to maintain the SAC. The prediction is that a single unattached kinetochore can generate enough new MCC to replenish the pool that is turned over by the APC/C and p31^{comet}/TRIP13, although how such sensitivity is achieved remains unclear. One possibility is that MCC turnover is restrained during mitosis by the action of deubiquitinating enzymes (DUBs), and USP44 is one such enzyme believed to antagonize Cdc20 ubiquitination to strengthen the SAC (Stegmeier et al., 2007).

We show here that another DUB, USP9X, plays an important role in the SAC. USP9X does not influence kinetochore signaling, but rather it restrains Cdc20 ubiquitination, degradation, and MCC turnover to maintain effective inhibition of the APC/C in the presence of unattached kinetochores. This function is critical for mitotic fidelity because loss of USP9X causes a reduction in the efficiency of the SAC, an increased frequency of chromosome mis-segregations and the generation of chromosomal instability (CIN). This could help explain why reduced USP9X

This is an open access article under the CC BY license (http://creativecommons.org/licenses/by/4.0/).

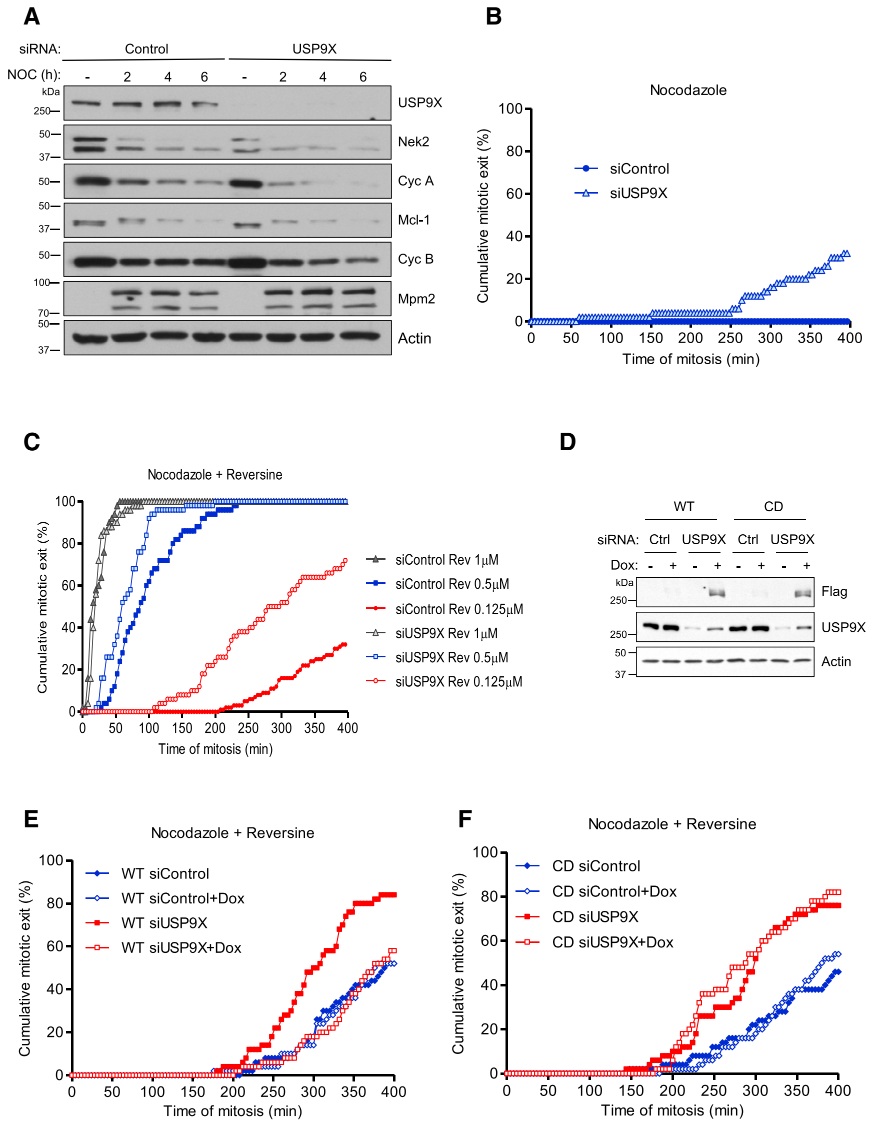

Figure 1. USP9X Catalytic Activity Is Required for an Efficient SAC Arrest

(A) Western blot analysis with indicated antibodies to show degradation of APC/C substrates during a mitotic arrest induced by nocodazole (100 ng/mL) in U2OS cells treated with control or USP9X siRNAs. Nocodazole-arrested cells were collected by wash-off and cultured for a further 2, 4, or 6 hr. Asynchronous (−) cells were transfected with indicated siRNA but not treated with nocodazole.

(B and C) Time-lapse analysis of duration of mitosis in U2OS cells treated with control or USP9X siRNAs and arrested with nocodazole (B) or nocodazole and different concentrations of reversine (C), as indicated. For time-lapse analysis, 50 cells were analyzed per condition, per experiment. All graphs shown are representative of three independent experiments.

(D) Western blot showing induction of siRNA-resistant FLAG-USP9X wild-type (WT) and catalytically dead (CD) protein after knockdown of endogenous USP9X in HeLa FRT cells. Cells were transfected with control or USP9X-1 siRNA for 24 hr, prior to addition of Dox for 48 hr to induce expression of Flag-USP9X. Samples were blotted with antibodies as indicated.

(E and F) Time-lapse analysis of duration of mitosis in HeLa FRT-Flag USP9X (E) catalytically active (WT) or (F) CD cells after knockdown of endogenous USP9X and induction of Flag-USP9X as in (D). Cells entered mitosis in the presence of nocodazole (250 ng/mL) and reversine (0.35 μM) and were imaged every 4 min. Fifty cells were analyzed per condition.

See also Figure S1.

expression has previously been linked to tumorigenesis in some tissues (Pereira et al., 2016; Pérez-Mancera et al., 2012; Thanh Nguyen et al., 2016).

RESULTS AND DISCUSSION

USP9X Is Required for an Efficient Mitotic Arrest

USP9X suppresses tumorigenesis in pancreatic ductal cancer, and its expression is reduced in several other cancers (Pereira et al., 2016; Pérez-Mancera et al., 2012; Thanh Nguyen et al., 2016). USP9X has, however, also been proposed to promote tumor cell survival by limiting the degradation of the anti-apoptotic protein Mcl-1 (Schwickart et al., 2010). Because Mcl-1 is a major determinant of cell fate in response to microtubule poisons (Harley et al., 2010; Sloss et al., 2016; Wertz et al., 2011), we sought to determine whether USP9X stabilized Mcl-1 during mitosis. Control or USP9X-depleted human osteosarcoma U2OS cells were arrested in mitosis with the microtubule-destabilizing drug nocodazole for 2, 4, or 6 hr to examine the rate of degradation of Mcl-1 and key mitotic regulators. Although USP9X depletion did not cause an obvious change in Mcl-1 degradation during mitosis, it did increase the loss of other mitotic APC/C substrates, in particular cyclin A, but also cyclin B and NEK2A (Figure 1A). Therefore, although USP9X does not have a specific role in controlling Mcl-1 during mitosis, it does appear to stabilize other APC/C substrates.

USP9X might function in mitosis to oppose the ubiquitination of certain APC/C substrates directly, or it might control their stability indirectly through an effect on the SAC that restrains APC/C activity. To test the possible role of USP9X in the SAC, we examined the ability of U2OS cells treated with nocodazole to maintain a prolonged mitotic arrest. We found that USP9X depletion reduced the duration of the arrest and cells exited mitosis despite the presence of the drug (Figure 1B). This effect was also apparent when nocodazole was combined with different doses of the Mps1 inhibitor reversine to reduce SAC strength in a stepwise manner (Santaguida et al., 2011; Saurin et al., 2011) (Figure 1C). The effect of USP9X depletion on mitotic arrest was also observed with a second small interfering RNA (siRNA)

targeting USP9X (Figure S1A). To investigate whether USP9X catalytic activity is required to maintain the duration of mitotic arrest, we generated HeLa-FRT cell lines stably expressing doxycycline (Dox)-inducible and siRNA-resistant wild-type (WT) USP9X or a catalytically dead (CD) form of USP9X in which the critical cysteine residue in the active site is mutated (C1566A) (Al-Hakim et al., 2008; Naik and Dixit, 2016) (Figure 1D). Following depletion of endogenous USP9X, the normal duration of mitotic arrest was restored by expression of WT-USP9X, showing that the effect of USP9X depletion by siRNA is specific and not an off-target effect (Figure 1E). Importantly expression of CD-USP9X failed to restore the timing of mitotic arrest, demonstrating that USP9X catalytic activity is required for this function (Figure 1F). Similar results were observed in cell lines derived from U2OS FRT cells in which WT- or CD-USP9X were expressed (Figures S1B–S1D). Collectively, these data demonstrate that USP9X catalytic activity is required for cells to maintain an efficient mitotic arrest in response to a microtubule poison that engages and sustains the SAC.

USP9X Acts Downstream of Kinetochores to Maintain the SAC

USP9X might help engage the SAC by acting through signaling components at the kinetochore to generate a stronger checkpoint signal. Alternatively, it might reinforce this checkpoint signal by acting further downstream, for example, to limit substrate ubiquitination by APC/C. To examine if USP9X affects SAC signaling at kinetochores, we quantified the kinetochore accumulation of SAC proteins in either control or USP9X-depleted cells arrested in mitosis with nocodazole. We observed no significant differences in the kinetochore levels of Mad1, Mad2, or BubR1, in either nocodazole alone, or following a 30 min incubation with nocodazole and MG132 with different doses of Mps1 inhibitor to modulate the kinetochore signal (Figures 2A–2F). Furthermore, baseline phosphorylation of the MELT motifs on KNL1, a crucial Mps1 substrate needed for the SAC (London et al., 2012; Shepperd et al., 2012; Yamagishi et al., 2012), was also unaffected by USP9X knockdown (Figures S2A and S2B). We conclude, therefore, that both Mps1 activity and kinetochore SAC signaling are unaffected by USP9X depletion. We confirmed that USP9X depletion affected the efficiency of the checkpoint under these conditions by quantifying the time to mitotic exit when different doses of Mps1 inhibitor were applied to nocodazole-arrested cells. Figure 2G shows that mitotic exit upon Mps1 inhibition was accelerated by USP9X depletion even though kinetochore signaling is unaffected under identical conditions (Figures 2A–2F).

USP9X has previously been reported to affect the levels of survivin, a key component of the chromosomal passenger complex that controls the activity of aurora B kinase (Vong et al., 2005). This could potentially affect SAC strength, because aurora B is known to affect the SAC directly (Santaguida et al., 2011; Saurin et al., 2011) and also indirectly by inhibiting kinetochore-microtubule attachments (Lampson et al., 2004). However, a number of lines of evidence suggest that this is not the case: first, we observed no difference upon USP9X depletion in kinetochore/centromere levels of aurora B or aurora B-pT232, a marker for active aurora B (Yasui et al., 2004) (Figures S2C–S2F); second, we observed no changes in the kinetochore levels of Mad2/BubR1 (Figures 2C–2F), which are known to be reduced upon aurora B inhibition (Ditchfield et al., 2003); and, third, we observed no reduction in the levels of survivin following USP9X knockdown (Figure S2G). We conclude therefore that the SAC phenotype upon USP9X knockdown is unrelated to survivin or aurora B activity.

Considering that USP9X depletion had no effect on the level of signaling components at the kinetochore, we hypothesized that USP9X might function instead to reinforce the SAC downstream of kinetochores. To test this, we sought to use a condition in which the SAC could be engaged in the absence of kinetochore signaling: we chose to use YFP-Mad2 overexpression, because this fusion protein can bind to MCC (presumably because of a stable "closed" Mad2 conformation) and induce a prolonged metaphase arrest due to constitutive SAC engagement in the absence of kinetochore signaling (Tipton et al., 2011, 2013). As expected, Dox-induced expression of YFP-Mad2 caused a prolonged mitotic arrest, which was completely independent of kinetochore signaling because it was unaffected by Mps1 inhibition with a high dose of either AZ 3146 (Hewitt et al., 2010) or reversine (Figure 2H). Importantly, this arrest was still severely attenuated by two separate siRNAs targeting USP9X (Figure 2H). Furthermore, it was not due to differences in the level of YFP-Mad2 overexpression or changes in the expression of other components of the MCC, which all remained unaltered (Figures 2I and S2H). Collectively, these data strongly support the conclusion that USP9X acts downstream of kinetochores to strengthen a SAC-dependent arrest.

USP9X Restrains APC/C Activity toward SAC-Controlled Substrates

Because USP9X is a DUB and the SAC signal relies on inhibition of the APC/C ubiquitin ligase, USP9X might antagonize APC/C-dependent ubiquitination to help maintain the SAC. USP9X could potentially reinforce the SAC in two ways: it could limit ubiquitination and degradation of certain key APC/C substrates, therefore reducing the requirement for APC/C inhibition by the MCC, or it could inhibit ubiquitination and turnover of the MCC itself (Musacchio and Ciliberto, 2012), thus increasing the strength of APC/C inhibition. To examine these possibilities, we monitored the degradation of several APC/C substrates using fluorescent reporters in live cells. We first examined the degradation of cyclin B1, cyclin A, and NEK2A arrested in mitosis by nocodazole because these APC/C substrates are all known to be influenced by the strength of the SAC (Boekhout and Wolthuis, 2015; Brito and Rieder, 2006; Collin et al., 2013). In each case, we monitored degradation in the presence or absence of USP9X and/or reversine at 0.25 μM to partially inhibit Mps1. When the SAC was weakened by reversine, the degradation of cyclin B, cyclin A, and NEK2A was accelerated, as shown previously (Boekhout and Wolthuis, 2015; Collin et al., 2013) (Figures 3A–3C). Importantly, USP9X depletion was also sufficient to accelerate the degradation of all of these substrates, particularly in the absence of reversine when the rate of degradation was under restraint by the SAC (Figures 3A–3C). These data demonstrate that USP9X controls the rate of degradation of SAC-controlled APC/C substrates during a mitotic arrest.

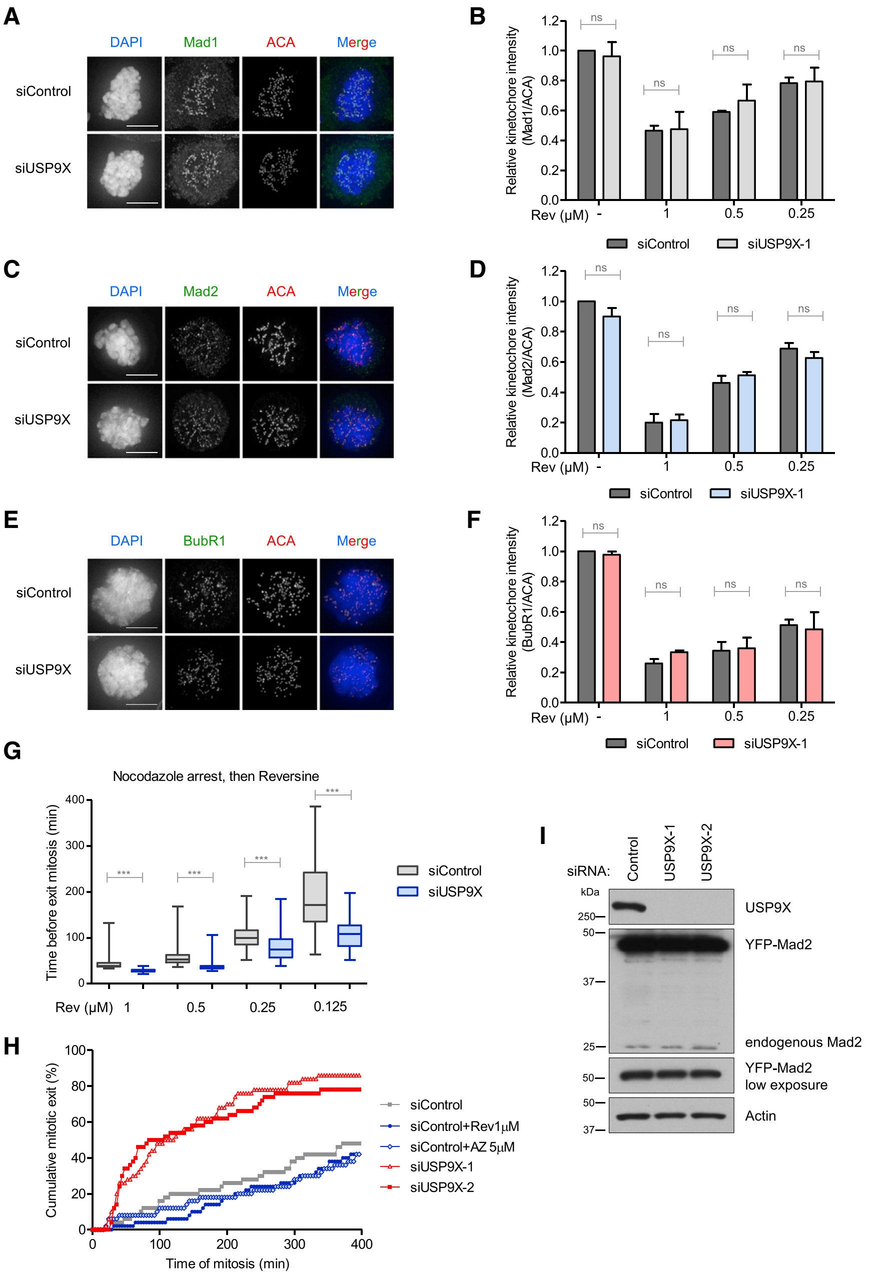

Figure 2. USP9X Controls the SAC Downstream of Kinetochores

(A–F) Representative images (A, C, and E) and quantification (B, D, and F) of Mad1 (A and B), Mad2 (C and D), and BubR1 (E and F) localization to kinetochores in nocodazole-arrested U2OS cells treated with control or USP9X siRNAs. ACA was used as a kinetochore marker. Cells were treated with nocodazole for 4 hr, before DMSO or reversine was added for 30 min, together with proteasome inhibitor MG132 to prevent mitotic exit. The graphs in (B), (D), and (F) show the mean kinetochore intensities (±SD) relative to mock-transfected, DMSO-treated cells, from three independent experiments with ten cells quantified for each condition per experiment. ns, not significant, unpaired Student's t test. Scale bar, 10 µM.

(G) Quantification of the time to mitotic exit for nocodazole-arrested U2OS cells treated with control or USP9X siRNAs that were subsequently treated with the indicated doses of reversine to inhibit the kinetochore signal. The box-and-whisker plots show the median (bar) together with minima and maxima (whiskers). The data are from three independent experiments with 50 cells quantified for each condition per experiment. $^{***}p < 0.001$, unpaired Student's t test.

(H) Time-lapse analysis of duration of mitotic arrest in U2OS cells treated with control or USP9X siRNAs and induced to express YFP-Mad2 for 16 hr prior to imaging. Cells were treated with reversine (1 µM) or AZ-3146 (5 µM) prior to imaging, where indicated, to abolish the kinetochore SAC signal. Fifty cells were analyzed per condition, and the graph is representative of three experiments.

(I) Western blot analysis of U2OS cells, treated with control or USP9X siRNA, and induced to express YFP-Mad2 for 16 hr.

See also Figure S2.

We next sought to address whether USP9X could also affect the degradation of APC/C substrates not controlled by the SAC. We therefore monitored KIF18A, an APC/C^{Cdc20} substrate that is degraded early in anaphase following SAC silencing (Sedgwick et al., 2013). As expected, KIF18A was not degraded during a nocodazole arrest (Figure S3A), but it was degraded shortly after mitotic exit following an unperturbed mitosis (Figure 3D). The rate of degradation of KIF18A during anaphase was unaffected by USP9X depletion (Figure 3D), although we could not exclude the possibility that USP9X activity is lost during anaphase, when KIF18A is degraded. To circumvent this issue, we next examined the degradation of Mcl-1, which is not affected by MCC levels or the SAC although its mitotic degradation is APC/C-dependent (L.A.A., unpublished data). Consistent with a lack of sensitivity to the checkpoint, we found that Mcl-1 degradation during a mitotic arrest in nocodazole was unaffected by Mps1 inhibition and/or USP9X depletion (Figure 3E), even though it is inhibited by APC11 knockdown (Figure S3B). This confirms our earlier observation (Figure 1A) that USP9X is apparently not a DUB for Mcl-1 during mitosis, and it also suggests that it is not a general DUB for all APC/C substrates.

Because Mcl-1 is an atypical APC/C substrate, we also tested whether the mitotic degradation of a canonical APC/C^{Cdc20} substrate, cyclin B, was affected by USP9X depletion. To prevent any effects on MCC levels from indirectly affecting cyclin B degradation, we monitored cyclin B levels in BubR1-depleted cells treated with 1 µM reversine to abolish the SAC. Under these conditions, cyclin B was degraded immediately following nuclear envelope breakdown, and the rate of degradation was

OPEN
ACCESS
CellPress

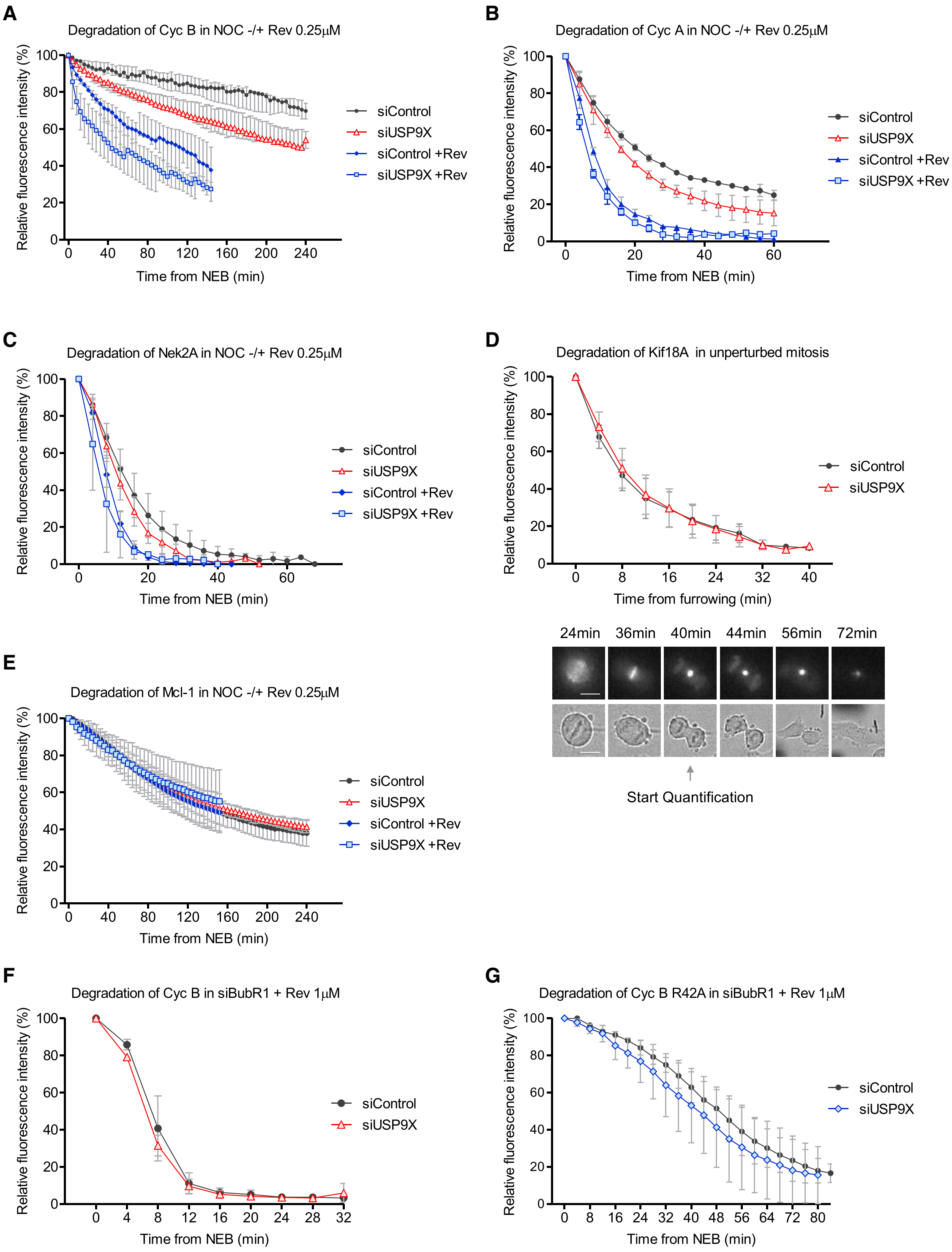

Figure 3. USP9X Restrains APC/C Activity toward SAC-Dependent Substrates

(A) Time-lapse analysis of endogenous Cyclin B-eYFP degradation in U2OS cells treated with control or USP9X siRNAs and arrested in nocodazole, with or without reversine (0.25 μM), as indicated.

(B and C) Time-lapse analysis of cyclin A-Venus (B) or YFP-Nek2A (C) degradation in HeLa cells treated as in (A).

(D) Time-lapse analysis of Venus-Kif18A degradation during an unperturbed mitosis in HeLa cells treated with control or USP9X siRNAs. Cell images below the graph indicate the point in anaphase when quantification started.

(E) Time-lapse analysis of YFP-Mcl-1 degradation in HeLa cells treated as in (A). Scale bar, 10 μM.

(legend continued on next page)

unaffected by USP9X knockdown (Figures 3F and S3C). To reduce the rate of cyclin B degradation, and to reveal potential differences when USP9X is knocked down, we analyzed the degradation of cyclin B-R42A, which carries a mutation within the D-box and is degraded with much slower kinetics than the WT protein (da Fonseca et al., 2011). We observed, however, only a slight effect of USP9X depletion on the rate of cyclin B-R42A degradation under these conditions (Figures 3G), in contrast to the pronounced effect on the degradation of cyclin B when the checkpoint was either fully or partially engaged (Figure 3A). We therefore conclude that USP9X specifically preserves the levels of MCC-dependent APC/C substrates during a mitotic arrest, which is likely to be due to enhanced MCC-mediated APC/C inhibition. Because USP9X functions downstream of kinetochores, we next examined its role on MCC turnover.

USP9X Restricts APC/C-Mediated MCC Turnover

The MCC itself is an APC/C substrate that is constitutively ubiquitinated and disassembled during mitosis (Eytan et al., 2013; Ge et al., 2009; Mansfeld et al., 2011; Reddy et al., 2007; Uzunova et al., 2012; Zeng et al., 2010). Although unattached kinetochores are present to catalyze MCC production, the rate of MCC assembly is thought to outstrip the rate of disassembly and the APC/C remains inhibited. Upon full bipolar attachment at metaphase, MCC production is shut down, and positive feedback drives rapid APC/C activation as APC/C degrades and disassembles its own inhibitor (Musacchio and Ciliberto, 2012). This ability of APC/C to release itself from MCC-mediated inhibition is dependent on APC/C activity (Ge et al., 2009; Reddy et al., 2007; Zeng et al., 2010) and on the APC15 subunit in particular, which specifically drives APC/C-mediated MCC turnover (Alfieri et al., 2016; Foster and Morgan, 2012; Mansfeld et al., 2011; Uzunova et al., 2012). We therefore reasoned that if USP9X antagonizes this process, then USP9X loss might reverse the mitotic delay caused by reduced levels of key APC/C subunits.

We first depleted the RING finger (APC11) or cullin (APC2) subunits that are critical for APC/C activity (Figures S4A and S4B). Either of these depletions caused a delay in mitotic exit, as expected, and this delay was indeed overcome by USP9X co-depletion (Figures 4A and S4C). The primary effect of APC11 or APC2 depletion therefore appears to be a strengthening of the SAC, and this is reversed by the loss of USP9X. In agreement with this conclusion, APC2-depleted cells could be induced to exit rapidly from a metaphase-arrested state by Mps1 inhibition with reversine (Figure S4D). Therefore, enough APC/C activity remains following APC2 depletion to promote mitotic exit, as long as the SAC is weakened by either Mps1 inhibition or USP9X depletion. We presume that residual APC/C activity following APC2 or APC11 depletion is due to the incomplete penetrance of siRNA.

We next targeted the APC15 subunit, because this does not affect APC/C activity per se but does inhibit APC/C^{MCC} turnover directly (Alfieri et al., 2016; Mansfeld et al., 2011; Uzunova et al., 2012). APC15 knockdown also caused a delay in mitosis that was abolished by co-depletion of USP9X (Figures 4B and S4E). The delay in metaphase following APC15 knockdown is due to a reduction in APC/C^{MCC} disassembly, which interferes with APC/C^{Cdc20} reactivation and subsequent cyclin B degradation (Mansfeld et al., 2011; Uzunova et al., 2012). To test the possibility that USP9X depletion rescues APC/C^{MCC} disassembly, we monitored the interaction of MCC with APC/C under similar conditions. Because of the requirement for large quantities of cells in these experiments, we generated a constitutive USP9X knockout cell line using CRISPR/Cas9 gene editing (Figure S4F), and we verified that the knockout cells had a weakened SAC response similar to that seen in cells depleted of USP9X by siRNA (Figure S4G) and that this was rescued by expression of WT- but not CD- USP9X (Figure S4H). Figures 4C and 4D show that the release of MCC from the APC/C was delayed by APC15 knockdown, as expected (Alfieri et al., 2016; Foster and Morgan, 2012; Mansfeld et al., 2011; Uzunova et al., 2012). However, this effect was diminished in USP9X knockout cells. Notably, knockout of USP9X itself did not alter the levels of APC15 protein (Figure S4I). We conclude, therefore, that APC/C-mediated turnover of the MCC is indeed restrained during mitosis by the catalytic activity of USP9X, which helps preserve an efficient SAC arrest.

APC/C^{Cdc20} disassembles MCC by ubiquitinating and degrading MCC-bound Cdc20 (MCC^{Cdc20}) in a process that requires APC15 (Alfieri et al., 2016; Foster and Morgan, 2012; Mansfeld et al., 2011; Uzunova et al., 2012). We hypothesized that USP9X might antagonize this ubiquitination to limit MCC turnover by APC/C. In agreement with this hypothesis, Cdc20 ubiquitination was enhanced during a mitotic arrest in USP9X knockout cells, even though steady-state levels of other MCC components remained unaltered (Figures 4E, 4F, and S4J). Endogenous Cdc20 is continuously synthesized during mitosis to replace the pool that is degraded by APC/C (Liang et al., 2012; Nilsson et al., 2008). We therefore monitored the levels of ectopically expressed Venus-Cdc20 in nocodazole-arrested cells, treated with control or USP9X siRNA. As expected, Cdc20 was degraded during a nocodazole arrest (King et al., 2007; Nilsson et al., 2008; Pan and Chen, 2004), but the rate of Cdc20 degradation was enhanced following USP9X depletion (Figure 4G). Importantly, this effect was in stark contrast to the reduction in the rate of Cdc20 degradation when cells were treated with reversine (Figure 4H), which is consistent with the effect of Mad2 depletion shown previously by Nilsson et al. (2008). Therefore, although weakening the kinetochore SAC signal slows the rate of Cdc20 degradation due to reduced

(F) Time-lapse analysis of endogenous cyclin B-eYFP degradation in control or USP9X siRNA-treated U2OS cells without a functional SAC. The SAC was abolished by BubR1 depletion and incubation with a high dose of reversine (1 μM).

(G) Time-lapse analysis of cerulean-cyclin B-R42A degradation in U2OS cells, treated as in (F). Doxycycline was added to express cyclin B-R42A for 44 hr prior to imaging.

All graphs in (A)–(G) show the mean values (±SD) from three independent experiments with ten cells quantified for each condition per experiment. NEB, nuclear envelope breakdown.

See also Figure S3.

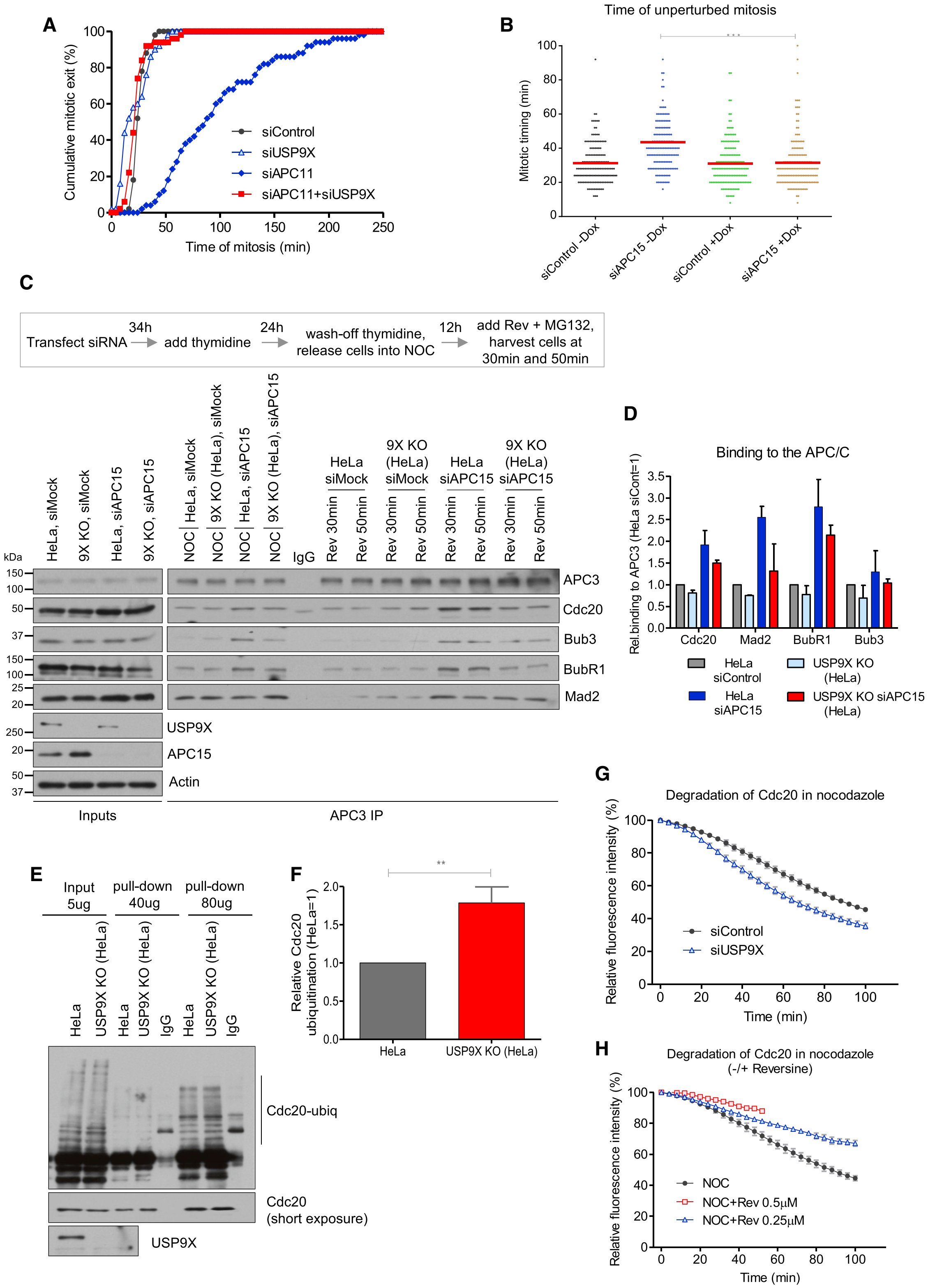

Figure 4. USP9X Restricts APC/C-Mediated Cdc20 Degradation and MCC Turnover

(A) Time-lapse analysis of duration of mitotic arrest in U2OS cells treated with indicated siRNAs. The graph shows cumulative data from at least 50 cells from one experiment, which is representative of three independent experiments.

(legend continued on next page)

MCC^{Cdc20} production, USP9X depletion actually enhances Cdc20 degradation by promoting MCC^{Cdc20} turnover. Collectively, these data demonstrate that USP9X restrains Cdc20 ubiquitination and degradation to limit MCC turnover and preserve a SAC arrest. We hypothesize that USP9X deubiquitinates Cdc20 directly; however, we cannot rule out the possibility that USP9X loss enhances Cdc20 ubiquitination and degradation indirectly. Biochemical analysis with purified components will be needed to resolve this issue.

USP9X Enhances SAC Efficiency to Protect against CIN

In the presence of many unattached kinetochores, the SAC signal is strong and therefore, although USP9X depletion reduces SAC efficiency in cells arrested in mitosis with nocodazole, the arrest still persists for several hours (Figure 1B). We hypothesized, however, that cells would rely more heavily on USP9X to restrain MCC turnover and preserve the SAC in the presence of just a few unattached kinetochores, when MCC production is at its lowest. To test this idea, we used the microtubule-stabilizing drug Taxol (paclitaxel) instead of nocodazole, because Taxol is known to arrest cells with fewer unattached kinetochores (Collin et al., 2013; Yang et al., 2009). We chose a dose that would mimic the limited mitotic arrest caused by just a single unattached kinetochore, estimated to be 2–3 hr in HeLa cells (Dick and Gerlich, 2013). Figure 5A shows that under these conditions, USP9X depletion reduced the Taxol-dependent arrest from 154 ± 21 to 98.8 ± 10.8 min.

We reasoned that this premature mitotic slippage due to a weakened SAC could lead to elevated levels of chromosome mis-segregations and enhanced CIN. To test this hypothesis, we generated multiple different early passage clones from USP9X knockout or control parental cells (Figure S5). We monitored chromosome segregation in these cells as they progressed through an unperturbed mitosis and observed an increase in the percentage of cells undergoing anaphase with misaligned or lagging chromosomes following USP9X knockout (Figures 5B and 5C). Chromosome counts of USP9X knockout or parental cells that were either early passage or late passage showed that although chromosome numbers in the control population did not change appreciably over time, the chromosome numbers in all five USP9X knockout clones became more heterogeneous in the high passage cells, mainly because of an increased proportion of cells containing fewer chromosomes (Figure 5D). Thus, loss of USP9X results in chromosome segregation defects and an inability to maintain their modal chromosomal number over time. This study therefore provides clear evidence that USP9X loss can lead to a CIN phenotype, presumably due to a weakening of the SAC. Importantly, because the kinetochore SAC signal is unaffected by USP9X loss, this also suggests that kinetochore MCC production alone is not sufficient to maintain chromosomal stability; rather, it must be aided by the restraint of MCC turnover by USP9X.

The increased level of CIN might help explain why disruption of USP9X cooperates with oncogenic K-Ras to drive pancreatic ductal adenocarcinomas (PDAs) in mice and why low USP9X expression correlates with poor survival following surgery and an increased metastatic burden in advanced human PDA (Pérez-Mancera et al., 2012). Furthermore, it may also explain why USP9X levels are decreased in several other cancers (Pereira et al., 2016; Thanh Nguyen et al., 2016). It will be interesting to determine whether USP9X levels are inversely correlated with the level of numerical chromosomal aberrations in these tumors.

However, although USP9X depletion might contribute to tumorigenesis by elevating CIN, there are many other routes to CIN that do not require USP9X loss (Bastians, 2015). Our data suggest that targeting USP9X that is retained in such tumors may be an effective therapeutic approach because too much CIN is detrimental to tumor cell survival (Andor et al., 2016; Birkbak et al., 2011; Janssen et al., 2009, 2011; Kops et al., 2004; Silk et al., 2013; Weaver et al., 2007). In fact, recent evidence suggests that tumors may reduce APC/C activity to limit CIN below the threshold needed for viability; the rationale is that partial APC/C inhibition extends the duration of metaphase and gives time for cells to correct kinetochore attachment errors and cluster centrosomes (Sansregret et al., 2017). In this report, we show that USP9X depletion rescues the mitotic delays caused by APC/C inhibition (Figures 4A, 4B, and S4A–S4C), which suggests that USP9X inhibition may be an effective strategy to elevate CIN and promote cell death in tumors that rely on USP9X expression and/or reduced APC/C activity for survival. Furthermore, reduced APC/C activity has also been shown to promote chemo-resistance against anti-mitotic therapeutics (Sansregret et al., 2017; Thu et al., 2018; Wild et al., 2016). This is apparently due to the extension of mitosis, caused by inefficient APC^{MCC} turnover, which implies that USP9X inhibition could re-sensitize tumors to these chemotherapeutics as well.

In summary, the data presented here show that USP9X antagonizes Cdc20 auto-ubiquitination and degradation to limit the turnover of MCC complexes by APC/C. A similar role has been reported previously for USP44 (Stegmeier et al., 2007), which

(B) Quantification of mitotic timing in HeLa cells uninduced (−) or induced (+) to express USP9X short hairpin RNAs (shRNAs) and treated with control or APC15 siRNAs. Mitotic timing indicates the time from mitotic cell rounding to anaphase. The graph shows the mean values (±SD) from three independent experiments with 50 cells quantified for each condition per experiment. ***$p < 0.001$, unpaired Student's t test.

(C and D) Western blot (C) and quantification (D) showing levels of MCC proteins co-precipitated with APC3 in lysates from control or USP9X knockout HeLa cells, with and without APC15 depletion, treated as indicated in panel inset. The graph in (D) shows the mean values (±SD) from at least two independent experiments for nocodazole-arrested cells treated with reversine and MG132 for 50 min.

(E and F) Western blot (E) and quantification (F) showing levels of Cdc20 ubiquitination precipitated with Cdc20 antibody in lysates from control or USP9X knockout HeLa cells, treated with nocodazole. The graph in (F) shows the mean values (±SD) from three independent experiments. **$p < 0.01$, unpaired Student's t test.

(G and H) Time-lapse analysis of Venus-Cdc20 degradation in nocodazole-arrested HeLa cells treated with either control or USP9X siRNAs (G), or different concentrations of reversine (H), as indicated. The graphs show the mean values (±SD) from three independent experiments with ten cells quantified for each condition per experiment.

See also Figure S4.

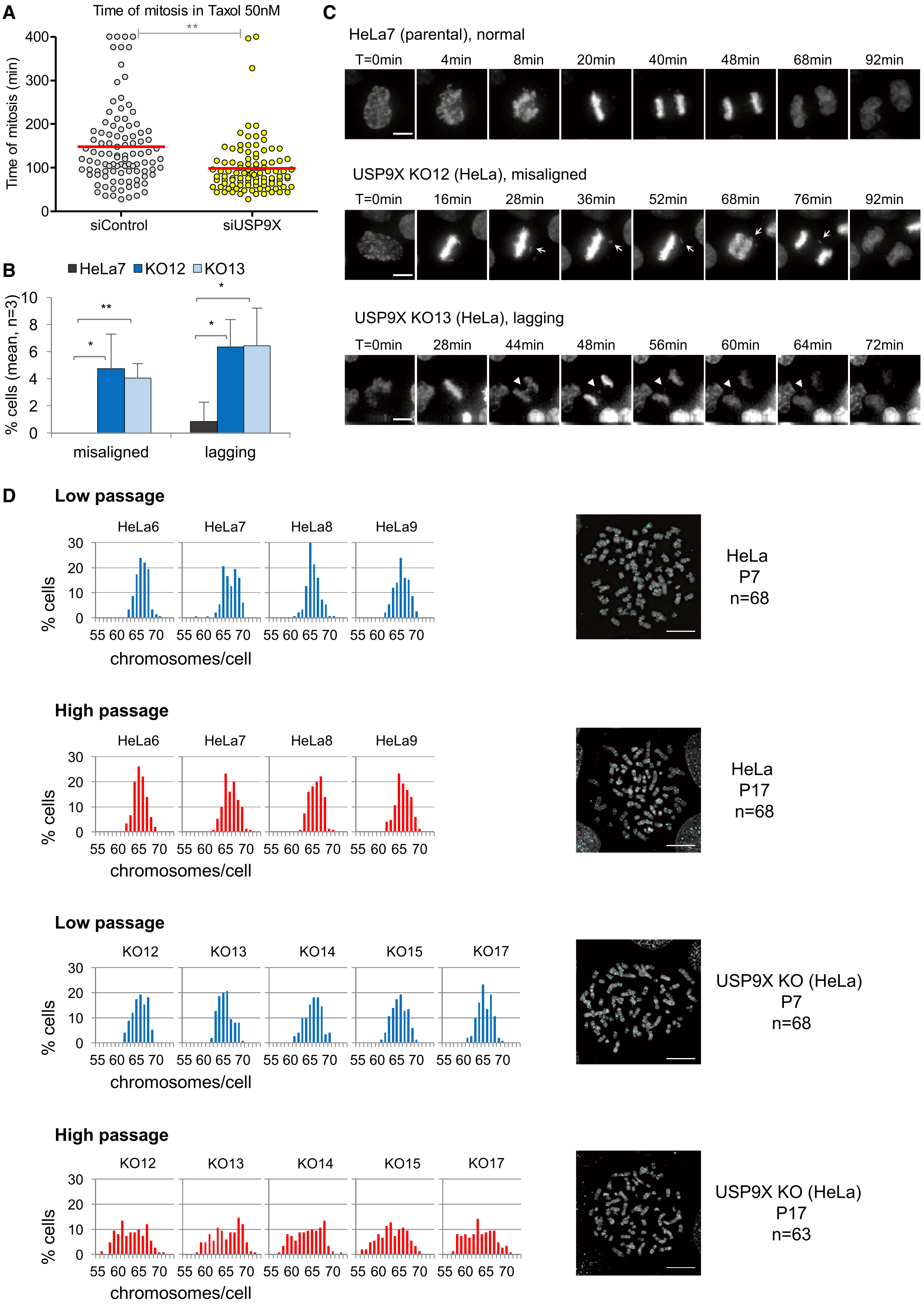

(legend on next page)

indicates that several different DUBs may reinforce the SAC by limiting APC/C activity. How USP9X targets MCC^{Cdc20} is currently unclear, but it will be important to determine in future whether it binds to any APC/C or MCC subunits, and in particular whether there are any mechanistic links between USP9X and APC15, the APC/C subunit that promotes Cdc20 ubiquitination and MCC turnover (Alfieri et al., 2016; Foster and Morgan, 2012; Mansfeld et al., 2011; Uzunova et al., 2012).

Finally, it will also be important to determine whether USP9X is regulated during mitosis, because this may reveal new strategies to modulate USP9X activity. If APC/C inhibition is a common mechanism used by tumors to limit excessive CIN and generate resistance against anti-mitotic drugs, as suggested (Sansregret et al., 2017), then elevating APC/C activity in tumors by targeted USP9X inhibition could represent a promising therapeutic strategy.

EXPERIMENTAL PROCEDURES

Cell Lines, Vectors, and Cell Treatments

Cells were cultured in DMEM supplemented with 10% fetal bovine serum (FBS) and 50 μg/mL penicillin/streptomycin. HeLa (OHIO) and U2OS (HTB96) were obtained from Cell Services, Cancer Research UK London Research Institute. To generate cell lines expressing Dox-inducible YFP-Mcl-1, cyclin A-Venus, or YFP-Nek2A, HeLa Flp-In cells stably expressing a TetR (Tighe et al., 2008) were co-transfected with pOG44 (Invitrogen) and cDNAs cloned into pc5-LAP-YFP or pc5-LAP-Venus using FuGene HD (Promega). Stable polyclonal cell lines were selected in media supplemented with hygromycin (200 μg/mL). pcDNA5 vectors to generate Dox-inducible Venus-Kif18A and Venus-Cdc20 cell lines (Sedgwick et al., 2013) were a kind gift from Jakob Nilsson (University of Copenhagen). U2OS cells expressing Dox-inducible YFP-Mad2 or cyclin B-R42A-cerulean were generated by transfecting U2OS-TR cells (Saurin et al., 2011) as above prior to selection with Zeocin (100 μg/mL). Monoclonal cell lines were established by limiting dilution and clones selected that gave the highest level of expression (YFP-Mad2, clone 3; CycB-R42A-cerulean, clone 20). HeLa Flp-in and U2OS Flp-in cells expressing Dox-inducible Flag-USP9X siRNA resistant to USP9X-1 (J-006099-07; Dharmacon) were generated by transfection with pCDNA5-FLAG-USP9X siRes-WT or siRes-CD (C1566A) (DU58547 and DU58585; MRC Protein Phosphorylation and Ubiquitylation Unit, University of Dundee) using FuGene HD. Stable polyclonal cell lines were selected in media supplemented with hygromycin (200 μg/mL; Roche). Endogenously tagged CycB-eYFP in U2OS cells were generated previously (Akopyan et al., 2014).

HeLa cells expressing USP9X shRNA were generated by annealing synthesized primers and subsequent ligation into pSUPERIOR vector (Oligoengine). Resulting plasmids were transfected into Phoenix Ampho 239 cells, using FuGene HD, to produce lentivirus expressing a Dox-inducible short hairpin targeting USP9X (GAAAUAACUUCCUACCGAAttcaagagaUUCGGU AGGAAGUUA-UUUC). Twenty-four hours after transfection, Phoenix Ampho 293 cells were washed and placed into fresh medium. Forty-eight hours after transfection, lentivirus-containing medium was collected and filtered through a 0.45 μm filter before addition onto HeLa Flp-in cells. Cells were infected with virus twice, in 24 hr intervals, before they were washed and selected in puromycin-containing media for 3 days. Dox-inducible knockdown of USP9X was then confirmed in polyclonal cells by western blotting.

USP9X CRISPR-Cas9 knockout (KO) cell lines were generated according to the method developed by Sanjana et al. (2014). Briefly, guide RNA sequences were designed against exon 2 using the online CRISPR design tool (http://crispr.mit.edu). The complementary oligonucleotides for guide RNAs (CACCGTGCATTTCCACATACTGACT-3′ and 5′-AAACAGTCAGTATGTGG AAATGCAC-3′) were annealed and cloned into lentiCRISPRv2 vector (AddGene plasmid #52961). Resulting plasmids were co-transfected into 293T cells together with packaging plasmids pMD2 and vsvG, for lentivirus production. Twenty-four hours after transfection, 293T cells were washed and placed into fresh medium. Forty-eight hours after transfection, lentivirus-containing medium was collected and filtered through a 0.45 μm filter before addition onto HeLa Flp-in and U2OS Flp-in cells. Cells were infected with virus for 24 h before they were washed and selected in puromycin-containing media for 3 days. KO of USP9X in polyclonal cells was confirmed by western blotting, which showed the pool population displayed almost no USP9X protein (Figure S4E). Clonal analysis demonstrated this residual expression was due to the presence of a small population of cells containing reduced USP9X expression. Considering the issues of working with single-cell clones from an aneuploid cancer line, we performed the majority of experiments with the pooled cell population. For analysis of chromosome segregation defects and aneuploidy, single clones of parental HeLa-Flp-in Cas9 and USP9X KO cells were isolated by limiting dilution.

The protocol for synchronizing cells in the period of mitotic arrest with nocodazole was described previously (Harley et al., 2010).

Antibodies and Reagents

The following antibodies were used for western blotting: APC11 (1/500, ab57158), Mcl-1 (1/500, ab559027), Nek2A (1/500, ab610593), and $p31^{comet}$ (1/1,000, ab97777) (all from Abcam); APC3 (1/1,000, 610454) and cyclin A (1/1,000, 611269) (both from BD Biosciences); BubR1 (1/2,500, A300-386A; Bethyl Laboratories); APC2 (1/1,000, 12301S; Cell Signaling Technology); MPM2 (1/1,000, 05-368; Millipore); survivin (1/1,000, 2235; ProSci); USP9X (55054-1; Proteintech); APC15 (1/1,000, sc-398488), Cdc20 (1/1,000, sc-13162), and cyclin B (1/2,500, sc-752) (all from Santa Cruz Biotechnology); actin (1/2,500, A2066), Bub3 (1/500, HPA003601), Flag (1/8,000, F3165), Mad2 (1/1,000, PA5-21594), and USP9X (1/1,000, WH0008239M1) (all from Sigma-Aldrich).

The following primary antibodies were used for immunofluorescence: aurora B (1/1,000, AIM-1, 611082; BD Biosciences); phospho-Thr232-aurora B (1/2,000, 600-401-677; Rockland); BubR1 (1/1,000, A300-386A) and Mad2 (1/1,000, A300-301A; Bethyl Laboratories); Mad1 (1/1,000, MABE867; Millipore); Cenp-C (1/1,000, PD-030; Caltag Medsystems); ACA/CREST (1/1,000, FZ90C-CS1058; Europa Bioproducts); and KNL1-pMELT13/17 (1/2,000, Nijenhuis et al., 2014).

Secondary antibodies were Alexa Fluor 647 goat-anti-guinea pig, Alexa Fluor 488 donkey anti-rabbit, and Alexa Fluor 568 goat anti-mouse (Invitrogen) for immunofluorescence and anti-mouse/rabbit-HRP (Bio-Rad) for western blotting (all at 1/1,000).

Figure 5. Loss of USP9X Induces Segregation Defects and Chromosomal Instability

(A) Average mitotic duration in Taxol-arrested (50 nM) U2OS cells treated with control or USP9X siRNAs. The graph shows the mean values (±SD) from two independent experiments with 50 cells quantified for each condition. $^{**}p < 0.01$, unpaired Student's t test.

(B and C) Quantification (B) and representative images (C) of misaligned (arrows) and lagging (arrowheads) chromosomes in HeLa parental cells (HeLa7) and USP9X knock out clones (KO12, KO13). The graph shows the mean values (±SD) from three independent experiments. At least 230 cells were analyzed per cell line with >60 cells per experiment. DNA was visualized using SiR-DNA, and cells were imaged every 4 min. $^{*}p < 0.05$ and $^{**}p < 0.01$, unpaired Student's t test. Scale bar, 10 μM.

(D) Chromosome counts from metaphase spreads in parental (HeLa 6, 7, 8, 9) or USP9X knockout (KO12, KO13, KO14, KO15, KO17) HeLa clones at early (P7) and late (P17) passage. The combined data for 150 cells from three independent experiments are shown; >40 cells were analyzed per experiment. Representative images of metaphase spreads from parental and USP9X knockout cells at early and late passage are shown (right). Scale bar, 10 μM.

See also Figure S5.

Dox (used at 1 μg/mL), thymidine (2 mM), paclitaxel, and reversine were purchased from Sigma-Aldrich. MG132 (10 μM) was purchased from Tocris Bioscience and AZ-3146 from Axon. Nocodazole (Calbiochem) was routinely used at 250 ng/mL, unless otherwise stated. Karyomax Colcemid solution was purchased from Thermo Fisher Scientific, and SiR-DNA was from Tebu-Bio.

siRNA

To knockdown protein expression, cells were transfected with indicated siRNA at a final concentration of 10 nM using Lipofectamine RNAiMAX Reagent (Invitrogen), following the manufacturer's instructions. ON-TARGET plus siRNAs from Dharmacon were used unless otherwise stated. The following siRNA duplexes were used in this study: USP9X-1: 5′-ACACGAUGCUUUA GAAUUU-3′ (J-006099-07); USP9X-2: 5′- GAAAUAACUUCCUACCGAA-3′ (J-006099-09); USP9X-3: 5′- GUACGACGAUGUAUUCUCA-3′ (J-006099-08); APC11-1: 5′-UCUGCAGGAUGGCAUUUAA-3′; APC11-2: 5′-AAGAUUAA GUGCUGGAACG-3′ (Mansfeld et al., 2011; Eurofins-MWG); APC2-2: 5′-GAUCGUAUCUACAAC AUGC-3′ (J-003200-12); APC2-3: 5′-GACAUCAU CACCCUCUAU-3′ (J-003200-11); APC2-4: 5′-GAGAUGAUCCAGCGUC UGU-3′ (J-003200-10); APC15 5′-CGAGAUGAAUGACUACAAU-3′ (Izawa and Pines, 2015); BubR1: AGAUCCUGGCUAACUGUUC; luciferase GL2: 5′-CGUACGCGGAAUACUUCGA-3′ (Eurofins-MWG); and GAPDH: 5′-AUUC CAUGGCACCGUCAAG-3′ (Eurofins-MWG).

To obtain knockdowns of USP9X and APC15, cells were transfected for 72 hr and for knockdown of BubR1 for 48 hr. For knockdown of APC11 and APC2, siRNA duplexes were pooled at an equimolar ratio and used at a final concentration of 10 nM. Transfection was carried out for 48 hr.

Live-Cell Imaging

For time-lapse analysis of duration of unperturbed mitosis or mitotic arrest, cells were plated into 24-well plates. The time in mitosis was measured as the period from a cell rounding to flattening. All quantifications that were compared directly were performed within the same experiment. Control or USP9X knockdowns were carried out for 60–70 hr, and medium was replaced with Leibovitz L-15 medium (Sigma-Aldrich), supplemented with 10% FBS, 2 mM L-glutamine, and 50 μg/mL penicillin/streptomycin. Cells were imaged in a heated chamber (37°C) using an EC Plan-Neofluar 10×/0.3 objective on a Zeiss Axiovert 200M microscope. Images were acquired using a C4742-80-12AG camera (Hamamatsu) and μmanager software and were processed using ImageJ Fiji.

For time-lapse analysis of duration of mitotic arrest in U2OS cells partially depleted of APC11 or APC2 and co-depleted of USP9X, cells were transfected with control or USP9X siRNA, followed by transfection with APC11 or APC2 siRNA 24 hr later. Cells were synchronized with thymidine and released into fresh medium for 10 hr before being imaged as described above.

To study degradation of fluorescently labeled cyclin A, Nek2A, Mcl-1, Kif18A, and Cdc20 by live-cell time-lapse analysis, cells were plated into eight-well chamber slides (Ibidi). Control or USP9X knockdowns were carried out for 60–70 hr, and medium was replaced with Leibovitz L-15 medium (Sigma-Aldrich), supplemented with 10% FBS, 2 mM L-glutamine, 50 μg/mL penicillin/streptomycin, and 1 μg/mL Dox. After 4 hr, nocodazole (with DMSO or reversine) was added, and a further 4 hr later, cells were imaged in a heated chamber (37°C), using a DeltaVision Core or Elite microscope system equipped with a 40×/1.30 NA U Plan FLN objective using softWoRx software (Applied Precision). Images were acquired with a CoolSNAP HQ2 camera (Photometrics) and processed using softWoRx software and ImageJ Fiji. Stacks of five images at 6 μm intervals were taken at 4 min intervals for 16–18 hr. Maximum intensity projection of the fluorescent channels was performed using softWoRx software. Fluorescence intensities are depicted as a percentage of the value at the time of mitotic entry.

For live-cell imaging of endogenous Cyclin B-eYFP in USP9X and BubR1 co-depleted cells, cells were plated in eight-well chamber slides (Ibidi), transfected with control or USP9X siRNA, followed by transfection with BubR1 siRNA. Twelve hours later, cells were synchronized with thymidine and released into fresh medium for 4 hr, followed by addition of reversine (1 μM) and imaging after 4 hr as described above.

For time-lapse analysis of cyclin B-R42A-cerulean degradation, cells were transfected with control or USP9X siRNA, followed by transfection with BubR1 siRNA. Twelve hours later, Dox was added and cells were arrested in G1/S phase for 24 hr by addition of thymidine. Next, cells were released into fresh medium for 4 hr, followed by addition of reversine (1 μM), and imaged after 4 hr as described above.

To study the effect of Mps1 inhibition on mitotic duration following depletion of APC2, cells were transfected with siRNA for 24 hr before being arrested with thymidine (2 mM) for 24 hr. Cells were then released into L-15 medium containing SiR-DNA (100 nM) for 15 min before being cultured in L-15 for a further 6 hr prior to the start of imaging. Reversine (1 μM) was added 4 hr later.

Immunofluorescence

For immunofluorescence analysis of localization of checkpoint proteins at kinetochores, cells plated on 12 mm coverslips were pre-extracted with 0.1% Triton X-100 in PEM (100 mM PIPES [pH 6.8], 1 mM $MgCl_2$ and 5 mM EGTA) for 60 s before fixation with 4% paraformaldehyde in PBS for 15 min. Coverslips were washed three times with PBS and blocked with 3% BSA in PBS for 1 hr, incubated with primary antibodies overnight at 4°C, washed with PBS, and incubated with secondary antibodies and DAPI for 4 hr at room temperature. Coverslips were then mounted using antifade (ProLong; Molecular Probes). CREST/ACA and Cenp-C were used as kinetochores markers, as indicated. All fixed images were acquired on a deconvolution system (Deltavision Elite or Core), equipped with a 100×/1.40 NA U Plan S Apochromat objective (Olympus) using softWoRx software. All displayed images are maximum-intensity projections of deconvolved stacks. For quantification of immunostaining, all images of similarly stained experiments were acquired with identical illumination settings and analyzed using ImageJ. An ImageJ macro was used to threshold and select all kinetochores, using the DAPI and anti-kinetochore antibody channels. This was used to calculate the relative mean kinetochore intensity of various proteins as shown previously (Saurin et al., 2011).

For analysis of chromosome segregation defects, cells were plated onto eight-well chamber slides (Ibidi) in DMEM supplemented with 10% FBS and 50 μg/mL penicillin/streptomycin. Four hours prior to imaging, cells were incubated with 100 nM SiR-DNA in L-15 supplemented with 10% FBS, 2 mM L-glutamine, and 50 μg/mL penicillin/streptomycin for 10 min before further incubation in L-15 media without SiR-DNA. Cells were then imaged every 4 min using a Deltavision Core or Elite microscope as described for analysis of fluorescent protein degradation.

Immunoprecipitation

To quantify levels of MCC proteins on APC/C, parental and USP9X KO cells were transfected with control siRNA or APC15 siRNA for 34 hr, synchronized in thymidine, and then released into fresh medium containing nocodazole for 12 hr. At that time, mitotic cells were either treated with MG132 for 30 min or with MG132 and reversine (0.5 μM) for 30 or 50 min. Cells were lysed in immunoprecipitation (IP) buffer (20 mM Tris [pH 7.5], 150 mM NaCl, 10% glycerol, 1% Triton X-100, 2 mM EDTA, 50 mM NaF, 5 mM β-glycerophosphate, 1 mM PMSF, protease inhibitor cocktail), and a total of 0.5–1 mg of cleared extract was incubated with 2 μg of APC3 antibody (sc-9972; Santa Cruz Biotechnology) or control IgG antibody (anti-sc35, 556363; BD Biosciences) for 2–4 hr and then with protein G Dynabeads (Thermo Fisher Scientific) for 90 min at 4°C on a rotating wheel. Precipitates were washed three times for 5 min with lysis buffer followed by incubation in SDS buffer for 4 min. Immunoblotting was performed using standard protocol, except that anti-mouse light chain-specific HRP-conjugated antibody was used as the secondary antibody (1/2,500, AP200P; Millipore).

To quantify Cdc20 ubiquitination, parental and USP9X KO cells were treated with nocodazole for 4 hr. Mitotic cells were collected by mitotic wash-off, and Cdc20 precipitation was performed using Cdc20 antibody (sc-13162; Santa Cruz Biotechnology), following the same protocol, but extracts were incubated with antibodies overnight at 4°C. Immunoblotting was performed as above for APC3 immunoprecipitations.

Karyotype Analysis of Metaphase Spreads

Cells were treated with 150 ng/mL Colcemid solution for 30 min in DMEM supplemented with 10% FBS and 50 μg/mL penicillin/streptomycin. Cells

were collected by trypsinization and resuspended in hypotonic buffer (20 mM HEPES [pH 8.0], 1 mM $MgCl_2$, 20 mM KCl, and 0.2 mM $CaCl_2$) at room temperature for 10 min. Samples were transferred to glass slides using a cytospin, air-dried, fixed in methanol/acetic acid (3:1) at room temperature for 10 min, and then washed twice in PBS. Immunofluorescence using Cenp-C antibody and counterstaining with DAPI were carried out as described above. Imaging was carried out using a Deltavision Core or Elite microscope equipped with a 100×/1.40 NA U Plan S Apochromat objective (Olympus) and a CoolSNAP HQ2 camera (Photometrics) using softWoRx software. Stacks of 20 images at 0.2 μm intervals were taken. All images are projections of deconvolved stacks.

Statistical Analysis

Statistical analysis was performed using GraphPad Prism 5 software. Differences between samples were assessed using unpaired Student's t tests. p values < 0.05 were considered to indicate statistical significance.

SUPPLEMENTAL INFORMATION

Supplemental Information includes five figures and can be found with this article online at https://doi.org/10.1016/j.celrep.2018.03.100.

ACKNOWLEDGMENTS

This study was funded by Cancer Research UK (C275/A13424 to P.R.C. and C47320/A21229 to A.T.S.) and Worldwide Cancer Research (formerly the Association for International Cancer Research; 12-0105 to P.R.C.). Microscopy was carried out at the Dundee Imaging Facility. We are grateful to Jakob Nilsson (University of Copenhagen) for providing cell lines.

AUTHOR CONTRIBUTIONS

A.S. and L.A.A. carried out the experiments and analyzed the data. All authors contributed to the manuscript. A.T.S. and P.R.C. co-supervised the study.

DECLARATION OF INTERESTS

The authors declare no competing interests.

Received: April 10, 2017
Revised: February 4, 2018
Accepted: March 21, 2018
Published: April 17, 2018

REFERENCES

Akopyan, K., Silva Cascales, H., Hukasova, E., Saurin, A.T., Müllers, E., Jaiswal, H., Hollman, D.A., Kops, G.J., Medema, R.H., and Lindqvist, A. (2014). Assessing kinetics from fixed cells reveals activation of the mitotic entry network at the S/G2 transition. Mol. Cell *53*, 843–853.

Al-Hakim, A.K., Zagorska, A., Chapman, L., Deak, M., Peggie, M., and Alessi, D.R. (2008). Control of AMPK-related kinases by USP9X and atypical Lys(29)/Lys(33)-linked polyubiquitin chains. Biochem. J. *411*, 249–260.

Alfieri, C., Chang, L., Zhang, Z., Yang, J., Maslen, S., Skehel, M., and Barford, D. (2016). Molecular basis of APC/C regulation by the spindle assembly checkpoint. Nature *536*, 431–436.

Andor, N., Graham, T.A., Jansen, M., Xia, L.C., Aktipis, C.A., Petritsch, C., Ji, H.P., and Maley, C.C. (2016). Pan-cancer analysis of the extent and consequences of intratumor heterogeneity. Nat. Med. *22*, 105–113.

Bastians, H. (2015). Causes of chromosomal instability. Recent Results Cancer Res. *200*, 95–113.

Birkbak, N.J., Eklund, A.C., Li, Q., McClelland, S.E., Endesfelder, D., Tan, P., Tan, I.B., Richardson, A.L., Szallasi, Z., and Swanton, C. (2011). Paradoxical relationship between chromosomal instability and survival outcome in cancer. Cancer Res. *71*, 3447–3452.

Boekhout, M., and Wolthuis, R. (2015). Nek2A destruction marks APC/C activation at the prophase-to-prometaphase transition by spindle-checkpoint-restricted Cdc20. J. Cell Sci. *128*, 1639–1653.

Brito, D.A., and Rieder, C.L. (2006). Mitotic checkpoint slippage in humans occurs via cyclin B destruction in the presence of an active checkpoint. Curr. Biol. *16*, 1194–1200.

Chao, W.C., Kulkarni, K., Zhang, Z., Kong, E.H., and Barford, D. (2012). Structure of the mitotic checkpoint complex. Nature *484*, 208–213.

Collin, P., Nashchekina, O., Walker, R., and Pines, J. (2013). The spindle assembly checkpoint works like a rheostat rather than a toggle switch. Nat. Cell Biol. *15*, 1378–1385.

Corbett, K.D. (2017). Molecular mechanisms of spindle assembly checkpoint activation and silencing. Prog. Mol. Subcell. Biol. *56*, 429–455.

da Fonseca, P.C., Kong, E.H., Zhang, Z., Schreiber, A., Williams, M.A., Morris, E.P., and Barford, D. (2011). Structures of APC/C(Cdh1) with substrates identify Cdh1 and Apc10 as the D-box co-receptor. Nature *470*, 274–278.

Dick, A.E., and Gerlich, D.W. (2013). Kinetic framework of spindle assembly checkpoint signalling. Nat. Cell Biol. *15*, 1370–1377.

Ditchfield, C., Johnson, V.L., Tighe, A., Ellston, R., Haworth, C., Johnson, T., Mortlock, A., Keen, N., and Taylor, S.S. (2003). Aurora B couples chromosome alignment with anaphase by targeting BubR1, Mad2, and Cenp-E to kinetochores. J. Cell Biol. *161*, 267–280.

Etemad, B., and Kops, G.J. (2016). Attachment issues: kinetochore transformations and spindle checkpoint silencing. Curr. Opin. Cell Biol. *39*, 101–108.

Eytan, E., Sitry-Shevah, D., Teichner, A., and Hershko, A. (2013). Roles of different pools of the mitotic checkpoint complex and the mechanisms of their disassembly. Proc. Natl. Acad. Sci. U S A *110*, 10568–10573.

Foster, S.A., and Morgan, D.O. (2012). The APC/C subunit Mnd2/Apc15 promotes Cdc20 autoubiquitination and spindle assembly checkpoint inactivation. Mol. Cell *47*, 921–932.

Ge, S., Skaar, J.R., and Pagano, M. (2009). APC/C- and Mad2-mediated degradation of Cdc20 during spindle checkpoint activation. Cell Cycle *8*, 167–171.

Harley, M.E., Allan, L.A., Sanderson, H.S., and Clarke, P.R. (2010). Phosphorylation of Mcl-1 by CDK1-cyclin B1 initiates its Cdc20-dependent destruction during mitotic arrest. EMBO J. *29*, 2407–2420.

Hewitt, L., Tighe, A., Santaguida, S., White, A.M., Jones, C.D., Musacchio, A., Green, S., and Taylor, S.S. (2010). Sustained Mps1 activity is required in mitosis to recruit O-Mad2 to the Mad1-C-Mad2 core complex. J. Cell Biol. *190*, 25–34.

Izawa, D., and Pines, J. (2015). The mitotic checkpoint complex binds a second CDC20 to inhibit active APC/C. Nature *517*, 631–634.

Janssen, A., Kops, G.J., and Medema, R.H. (2009). Elevating the frequency of chromosome mis-segregation as a strategy to kill tumor cells. Proc. Natl. Acad. Sci. U S A *106*, 19108–19113.

Janssen, A., Kops, G.J., and Medema, R.H. (2011). Targeting the mitotic checkpoint to kill tumor cells. Horm. Cancer *2*, 113–116.

King, E.M., van der Sar, S.J., and Hardwick, K.G. (2007). Mad3 KEN boxes mediate both Cdc20 and Mad3 turnover, and are critical for the spindle checkpoint. PLoS ONE *2*, e342.

Kops, G.J., Foltz, D.R., and Cleveland, D.W. (2004). Lethality to human cancer cells through massive chromosome loss by inhibition of the mitotic checkpoint. Proc. Natl. Acad. Sci. U S A *101*, 8699–8704.

Lampson, M.A., Renduchitala, K., Khodjakov, A., and Kapoor, T.M. (2004). Correcting improper chromosome-spindle attachments during cell division. Nat. Cell Biol. *6*, 232–237.

Liang, H., Lim, H.H., Venkitaraman, A., and Surana, U. (2012). Cdk1 promotes kinetochore bi-orientation and regulates Cdc20 expression during recovery from spindle checkpoint arrest. EMBO J. *31*, 403–416.

Lischetti, T., and Nilsson, J. (2015). Regulation of mitotic progression by the spindle assembly checkpoint. Mol. Cell. Oncol. *2*, e970484.

London, N., Ceto, S., Ranish, J.A., and Biggins, S. (2012). Phosphoregulation of Spc105 by Mps1 and PP1 regulates Bub1 localization to kinetochores. Curr. Biol. *22*, 900–906.

Luo, X., Tang, Z., Rizo, J., and Yu, H. (2002). The Mad2 spindle checkpoint protein undergoes similar major conformational changes upon binding to either Mad1 or Cdc20. Mol. Cell *9*, 59–71.

Mansfeld, J., Collin, P., Collins, M.O., Choudhary, J.S., and Pines, J. (2011). APC15 drives the turnover of MCC-CDC20 to make the spindle assembly checkpoint responsive to kinetochore attachment. Nat. Cell Biol. *13*, 1234–1243.

Musacchio, A. (2015). The molecular biology of spindle assembly checkpoint signaling dynamics. Curr. Biol. *25*, R1002–R1018.

Musacchio, A., and Ciliberto, A. (2012). The spindle-assembly checkpoint and the beauty of self-destruction. Nat. Struct. Mol. Biol. *19*, 1059–1061.

Naik, E., and Dixit, V.M. (2016). Usp9X is required for lymphocyte activation and homeostasis through its control of ZAP70 ubiquitination and PKCbeta kinase activity. J. Immunol. *196*, 3438–3451.

Nijenhuis, W., Vallardi, G., Teixeira, A., Kops, G.J., and Saurin, A.T. (2014). Negative feedback at kinetochores underlies a responsive spindle checkpoint signal. Nat. Cell Biol. *16*, 1257–1264.

Nilsson, J., Yekezare, M., Minshull, J., and Pines, J. (2008). The APC/C maintains the spindle assembly checkpoint by targeting Cdc20 for destruction. Nat. Cell Biol. *10*, 1411–1420.

Pan, J., and Chen, R.H. (2004). Spindle checkpoint regulates Cdc20p stability in Saccharomyces cerevisiae. Genes Dev. *18*, 1439–1451.

Pereira, B., Chin, S.F., Rueda, O.M., Vollan, H.K., Provenzano, E., Bardwell, H.A., Pugh, M., Jones, L., Russell, R., Sammut, S.J., et al. (2016). The somatic mutation profiles of 2,433 breast cancers refines their genomic and transcriptomic landscapes. Nat. Commun. *7*, 11479.

Pérez-Mancera, P.A., Rust, A.G., van der Weyden, L., Kristiansen, G., Li, A., Sarver, A.L., Silverstein, K.A., Grützmann, R., Aust, D., Rümmele, P., et al.; Australian Pancreatic Cancer Genome Initiative (2012). The deubiquitinase USP9X suppresses pancreatic ductal adenocarcinoma. Nature *486*, 266–270.

Pesenti, M.E., Weir, J.R., and Musacchio, A. (2016). Progress in the structural and functional characterization of kinetochores. Curr. Opin. Struct. Biol. *37*, 152–163.

Reddy, S.K., Rape, M., Margansky, W.A., and Kirschner, M.W. (2007). Ubiquitination by the anaphase-promoting complex drives spindle checkpoint inactivation. Nature *446*, 921–925.

Rieder, C.L., Cole, R.W., Khodjakov, A., and Sluder, G. (1995). The checkpoint delaying anaphase in response to chromosome monoorientation is mediated by an inhibitory signal produced by unattached kinetochores. J. Cell Biol. *130*, 941–948.

Sanjana, N.E., Shalem, O., and Zhang, F. (2014). Improved vectors and genome-wide libraries for CRISPR screening. Nat. Methods *11*, 783–784.

Sansregret, L., Patterson, J.O., Dewhurst, S., López-García, C., Koch, A., McGranahan, N., Chao, W.C.H., Barry, D.J., Rowan, A., Instrell, R., et al. (2017). APC/C dysfunction limits excessive cancer chromosomal instability. Cancer Discov. *7*, 218–233.

Santaguida, S., Vernieri, C., Villa, F., Ciliberto, A., and Musacchio, A. (2011). Evidence that Aurora B is implicated in spindle checkpoint signalling independently of error correction. EMBO J. *30*, 1508–1519.

Saurin, A.T., van der Waal, M.S., Medema, R.H., Lens, S.M., and Kops, G.J. (2011). Aurora B potentiates Mps1 activation to ensure rapid checkpoint establishment at the onset of mitosis. Nat. Commun. *2*, 316.

Schwickart, M., Huang, X., Lill, J.R., Liu, J., Ferrando, R., French, D.M., Maecker, H., O'Rourke, K., Bazan, F., Eastham-Anderson, J., et al. (2010). Deubiquitinase USP9X stabilizes MCL1 and promotes tumour cell survival. Nature *463*, 103–107.

Sedgwick, G.G., Hayward, D.G., Di Fiore, B., Pardo, M., Yu, L., Pines, J., and Nilsson, J. (2013). Mechanisms controlling the temporal degradation of Nek2A and Kif18A by the APC/C-Cdc20 complex. EMBO J. *32*, 303–314.

Shepperd, L.A., Meadows, J.C., Sochaj, A.M., Lancaster, T.C., Zou, J., Buttrick, G.J., Rappsilber, J., Hardwick, K.G., and Millar, J.B. (2012). Phosphodependent recruitment of Bub1 and Bub3 to Spc7/KNL1 by Mph1 kinase maintains the spindle checkpoint. Curr. Biol. *22*, 891–899.

Silk, A.D., Zasadil, L.M., Holland, A.J., Vitre, B., Cleveland, D.W., and Weaver, B.A. (2013). Chromosome missegregation rate predicts whether aneuploidy will promote or suppress tumors. Proc. Natl. Acad. Sci. USA *110*, E4134–E4141.

Sironi, L., Mapelli, M., Knapp, S., De Antoni, A., Jeang, K.T., and Musacchio, A. (2002). Crystal structure of the tetrameric Mad1-Mad2 core complex: implications of a 'safety belt' binding mechanism for the spindle checkpoint. EMBO J. *21*, 2496–2506.

Sloss, O., Topham, C., Diez, M., and Taylor, S. (2016). Mcl-1 dynamics influence mitotic slippage and death in mitosis. Oncotarget *7*, 5176–5192.

Stegmeier, F., Rape, M., Draviam, V.M., Nalepa, G., Sowa, M.E., Ang, X.L., McDonald, E.R., 3rd, Li, M.Z., Hannon, G.J., Sorger, P.K., et al. (2007). Anaphase initiation is regulated by antagonistic ubiquitination and deubiquitination activities. Nature *446*, 876–881.

Thanh Nguyen, H., Andrejeva, D., Gupta, R., Choudhary, C., Hong, X., Eichhorn, P.J., Loya, A.C., and Cohen, S.M. (2016). Deubiquitylating enzyme USP9x regulates hippo pathway activity by controlling angiomotin protein turnover. Cell Discov. *2*, 16001.

Thu, K.L., Silvester, J., Elliott, M.J., Ba-Alawi, W., Duncan, M.H., Elia, A.C., Mer, A.S., Smirnov, P., Safikhani, Z., Haibe-Kains, B., et al. (2018). Disruption of the anaphase-promoting complex confers resistance to TTK inhibitors in triple-negative breast cancer. Proc. Natl. Acad. Sci. U S A *115*, E1570–E1577.

Tighe, A., Staples, O., and Taylor, S. (2008). Mps1 kinase activity restrains anaphase during an unperturbed mitosis and targets Mad2 to kinetochores. J. Cell Biol. *181*, 893–901.

Tipton, A.R., Tipton, M., Yen, T., and Liu, S.T. (2011). Closed MAD2 (C-MAD2) is selectively incorporated into the mitotic checkpoint complex (MCC). Cell Cycle *10*, 3740–3750.

Tipton, A.R., Ji, W., Sturt-Gillespie, B., Bekier, M.E., 2nd, Wang, K., Taylor, W.R., and Liu, S.T. (2013). Monopolar spindle 1 (MPS1) kinase promotes production of closed MAD2 (C-MAD2) conformer and assembly of the mitotic checkpoint complex. J. Biol. Chem. *288*, 35149–35158.

Uzunova, K., Dye, B.T., Schutz, H., Ladurner, R., Petzold, G., Toyoda, Y., Jarvis, M.A., Brown, N.G., Poser, I., Novatchkova, M., et al. (2012). APC15 mediates CDC20 autoubiquitylation by APC/C(MCC) and disassembly of the mitotic checkpoint complex. Nat. Struct. Mol. Biol. *19*, 1116–1123.

Vong, Q.P., Cao, K., Li, H.Y., Iglesias, P.A., and Zheng, Y. (2005). Chromosome alignment and segregation regulated by ubiquitination of survivin. Science *310*, 1499–1504.

Weaver, B.A., Silk, A.D., Montagna, C., Verdier-Pinard, P., and Cleveland, D.W. (2007). Aneuploidy acts both oncogenically and as a tumor suppressor. Cancer Cell *11*, 25–36.

Wertz, I.E., Kusam, S., Lam, C., Okamoto, T., Sandoval, W., Anderson, D.J., Helgason, E., Ernst, J.A., Eby, M., Liu, J., et al. (2011). Sensitivity to antitubulin chemotherapeutics is regulated by MCL1 and FBW7. Nature *471*, 110–114.

Wild, T., Larsen, M.S., Narita, T., Schou, J., Nilsson, J., and Choudhary, C. (2016). The spindle assembly checkpoint is not essential for viability of human cells with genetically lowered APC/C activity. Cell Rep. *14*, 1829–1840.

Yamagishi, Y., Yang, C.H., Tanno, Y., and Watanabe, Y. (2012). MPS1/Mph1 phosphorylates the kinetochore protein KNL1/Spc7 to recruit SAC components. Nat. Cell Biol. *14*, 746–752.

Yamaguchi, M., VanderLinden, R., Weissmann, F., Qiao, R., Dube, P., Brown, N.G., Haselbach, D., Zhang, W., Sidhu, S.S., Peters, J.M., et al. (2016).

Cryo-EM of mitotic checkpoint complex-bound APC/C reveals reciprocal and conformational regulation of ubiquitin ligation. Mol. Cell *63*, 593–607.

Yang, Z., Kenny, A.E., Brito, D.A., and Rieder, C.L. (2009). Cells satisfy the mitotic checkpoint in Taxol, and do so faster in concentrations that stabilize syntelic attachments. J. Cell Biol. *186*, 675–684.

Yasui, Y., Urano, T., Kawajiri, A., Nagata, K., Tatsuka, M., Saya, H., Furukawa, K., Takahashi, T., Izawa, I., and Inagaki, M. (2004). Autophosphorylation of a newly identified site of Aurora-B is indispensable for cytokinesis. J. Biol. Chem. *279*, 12997–13003.

Zeng, X., Sigoillot, F., Gaur, S., Choi, S., Pfaff, K.L., Oh, D.C., Hathaway, N., Dimova, N., Cuny, G.D., and King, R.W. (2010). Pharmacologic inhibition of the anaphase-promoting complex induces a spindle checkpoint-dependent mitotic arrest in the absence of spindle damage. Cancer Cell *18*, 382–395.

Cell Reports, Volume *23*

Supplemental Information

USP9X Limits Mitotic Checkpoint Complex Turnover
to Strengthen the Spindle Assembly Checkpoint
and Guard against Chromosomal Instability

Agnieszka Skowyra, Lindsey A. Allan, Adrian T. Saurin, and Paul R. Clarke

Figure S1. SiRNA-mediated depletion of USP9X weakens the SAC, Related to Figure 1.

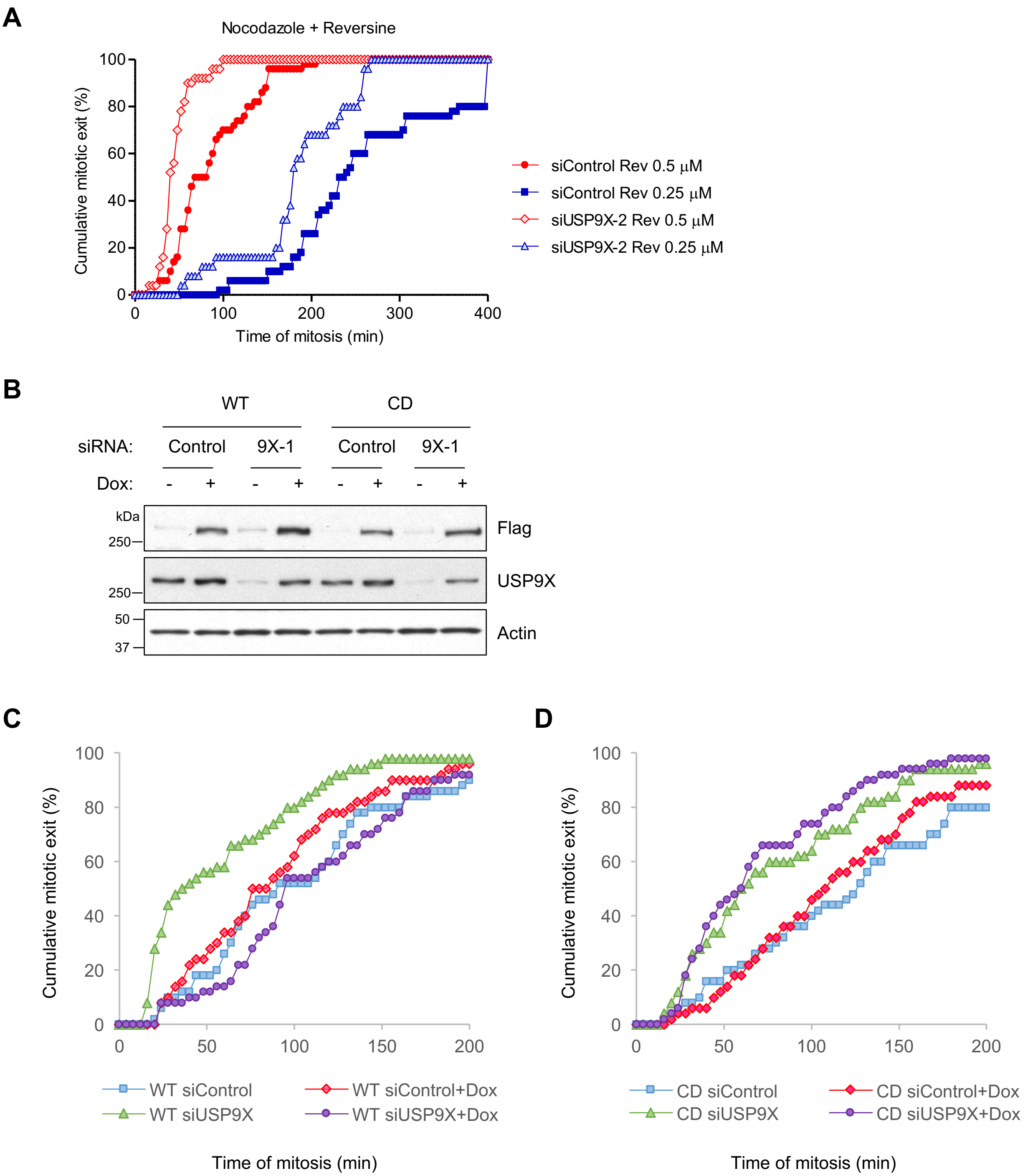

Figure S1. SiRNA-mediated depletion of USP9X weakens the SAC, Related to Figure 1.

A. Time-lapse analysis of duration of mitotic arrest in U2OS cells treated with control or USP9X-2 siRNAs and arrested in nocodazole with different concentrations of reversine, as indicated. 50 cells were analysed per condition, and graph is representative of 3 independent experiments.

B. Western blot showing induction of siRNA-resistant FLAG-USP9X wild type (WT) and catalytically dead (CD) protein after knockdown of endogenous USP9X in U2OS FRT cells. Cells were transfected with control or USP9X-1 siRNA for 24 h, prior to addition of Dox for 48 h to induce expression of Flag-USP9X. Samples were blotted with antibodies as indicated.

C,D. Time–lapse analysis of duration of mitosis in U2OS FRT cells expressing **(C)** Flag-USP9X wild type (WT) or (**D**) catalytically dead (CD) protein after knockdown of endogenous USP9X and induction of Flag-USP9X as in (**B**). Cells entered mitosis in the presence of nocodazole (250 ng/ml) and reversine (0.35 µM) and were imaged every 4 min. 50 cells were analysed per condition.

Figure S2. USP9X depletion does not affect the MPS1 or Aurora B activity at kinetochores, Related to Figure 2.

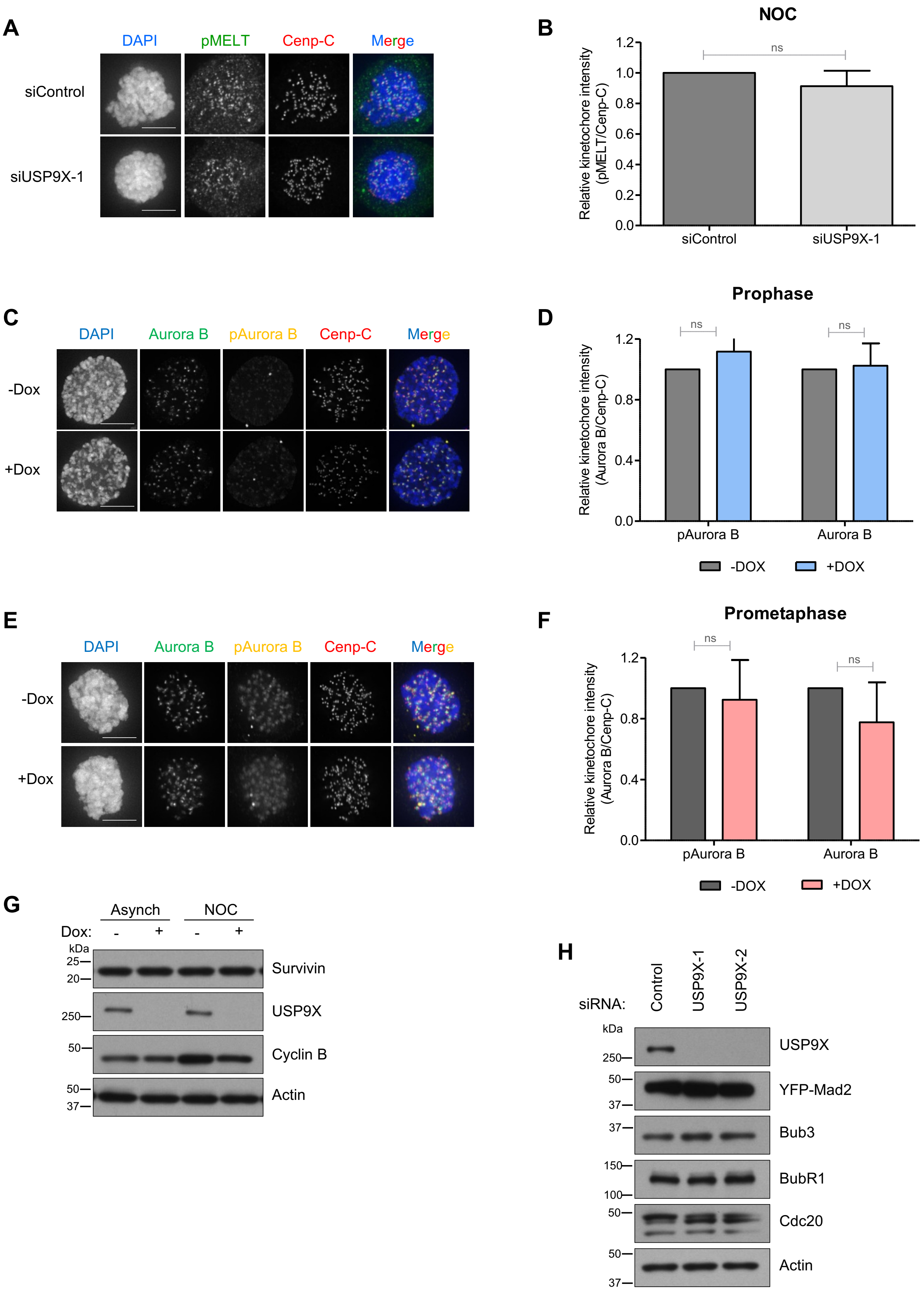

Figure S2. USP9X depletion does not affect the MPS1 or Aurora B activity at kinetochores, Related to Figure 2.

A,B. Representative images (A) and quantification (B) of KNL1-pMELT localization at kinetochores in nocodazole-arrested U2OS cells treated with control or USP9X siRNA. CENP-C was used as a kinetochore marker. Scale bar = 10 μM.

C-F. Representative images (C, E) and quantification (D, F) of phosphorylated Aurora B (Thr232) and total Aurora B localization at kinetochores during prophase (C, D) or prometaphase (E, F) in nocodazole-arrested HeLa cells that were uninduced (-Dox) or induced (+Dox) to express USP9X shRNAs.

All graphs show the mean kinetochore intensities (± S.D.), relative to mock-transfected cells, from 3 independent experiments with 10 cells quantified for each condition per experiment. ns, non-significant, Students unpaired t-test. Scale bar = 10 μM.

G. Western blot of whole-cell lysates, demonstrating equal levels of Survivin in cells treated as in (A).

H. Western blot of whole-cell lysates, demonstrating equal levels of MCC components in YFP-Mad2 expressing cells, transfected with control or USP9X siRNAs. Expression of YFP-Mad2 was induced 16 h before harvesting cells.

Figure S3. USP9X depletion does not affect the SAC-independent degradation of APC/C substrates, Related to Figure 3.

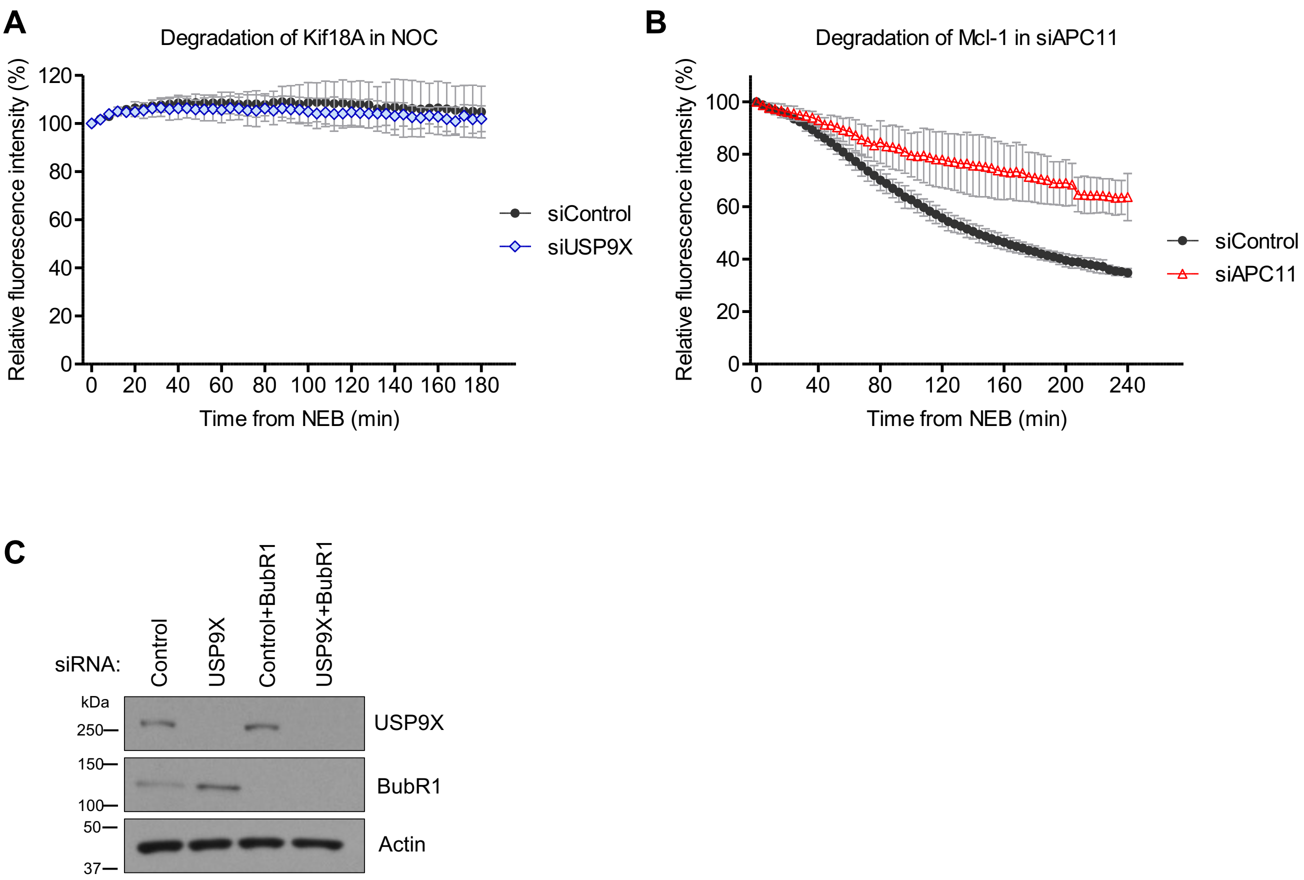

Figure S3. USP9X depletion does not affect the SAC-independent degradation of APC/C substrates, Related to Figure 3.
A,B. Time-lapse analysis of Venus-Kif18A degradation (A) or YFP-Mcl-1 degradation (B) in HeLa cells treated with control siRNA compared to either USP9X siRNA or APC11 siRNA, respectively, and arrested in mitosis with nocodazole. Graphs show the mean values (± S.D.) from 3 independent experiments with 10 cells quantified for each condition per experiment.
C. Western blot of whole-cell lysates of U2OS cells expressing endogenous Cyclin B-eYFP, demonstrating co-depletion of USP9X and BubR1.

Figure S4. Effect of USP9X depletion on APC/C-mediated CDC20 degradation and MCC turnover, Related to Figure 4.

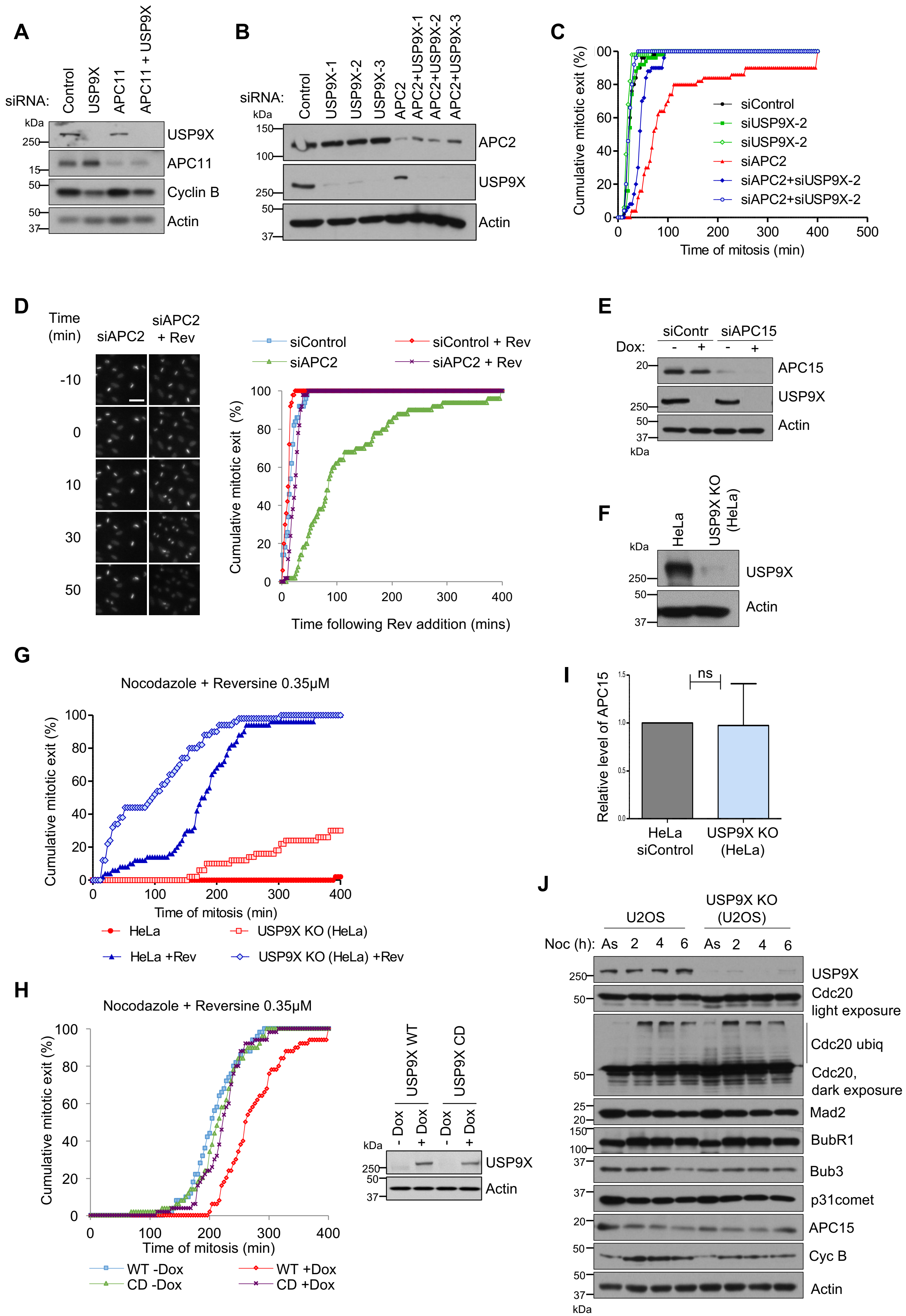

Figure S4. Effect of USP9X depletion on APC/C-mediated CDC20 degradation and MCC turnover, Related to Figure 4.

A. Western analysis demonstrating knockdown of USP9X and partial knockdown of APC11 in U2OS cells.

B. Western blot of whole-cell lysates from U2OS cells treated with indicated siRNAs, demonstrating knockdown of USP9X and partial knockdown of APC2.

C. Time-lapse analysis of duration of mitotic arrest in U2OS cells treated with indicated siRNAs. The graph shows cumulative data from at least 50 cells from one experiment, which is representative of 3 independent experiments.

D. Representative images and time-lapse analysis of duration of mitosis in HeLa-FRT cells transfected with control or APC2 siRNAs and allowed to arrest at metaphase. Cell were then either left untreated or treated with reversine (1µM) at t=0 min. The graph shows duration of mitosis following reversine addition. Scale bar = 50 µM.

E. Western analysis from HeLa cells, non-induced (-) or induced (+) to express USP9X shRNAs and treated with control or APC15 siRNAs, demonstrating APC15 knockdown is unaffected by USP9X co-depletion.

F. Western blot analysis demonstrating loss of USP9X protein in USP9X knockout cells compared with parental HeLa cells.

G. Time-lapse analysis of duration of mitotic arrest in control or USP9X knockout HeLa cells arrested in nocodazole with and without reversine (0.35 µM). The graph shows cumulative data from at least 50 cells from one experiment, which is representative of 2 independent experiments.

H. Time-lapse analysis of duration of mitotic arrest in HeLa USP9X knockout cells expressing doxycycline-inducible -wild-type (WT) or -catalytically dead (CD) USP9X. Cells were untreated or treated with Dox (1µg/ml) for 48h. Nocodazole (250ng/ml) and reversine (0.35µM) were added prior to imaging every 4 min. Dox-induction of WT- or CD- USP9X was analysed by western blotting. The graph shows cumulative data from at least 50 cells from one experiment, which is representative of 3 independent experiments.

I. Quantification of APC15 protein levels in parental and USP9X knockout (USP9X KO) HeLa cells using actin levels to control for loading. The graph shows the mean values (± S.D.) from 3 independent experiments. ns, non-significant, Students t-test.

J. Western blot analysis of MCC components and proteins involved in MCC turnover from control or USP9X knockout U2OS cells, either asynchronously growing (As) or arrested in nocodazole for indicated durations. Nocodazole-arrested cells were detached by shake off to exclude non-mitotic cells. Note increased ubiquitination of Cdc20 in nocodazole-arrested USP9X knockout (USP9X KO) cells compared to U2OS FRT cells with USP9X (U2OS).

Figure S5. Characterisation of HeLa-CRISPR-Cas9 USP9X knockout cells, Related to Figure 5.

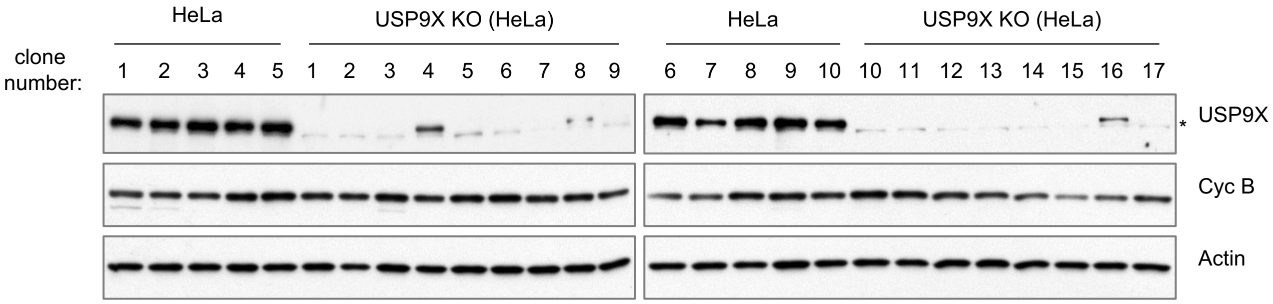

Figure S5. Characterisation of HeLa-CRISPR-Cas9 USP9X knockout cells, Related to Figure 5.
Western blotting showing absence of USP9X protein in USP9X knock-out (USP9X KO) single clones. Parental HeLa-Flp-in clones were analysed for comparison. * denotes a non-specific band detected by the USP9X antibody.
